# Public Health Communication Challenges in Eastern Europe and Central Asia: A Scoping Review

**DOI:** 10.3390/ijerph23010019

**Published:** 2025-12-22

**Authors:** Lisa Lim, Aisha Mukasheva, Augustina Osaromiyeke Alegbe, Adaora Nancy Emehel, Bibigul Aubakirova, Yuliya Semenova

**Affiliations:** 1 Graduate School of Public Policy, Nazarbayev University, Astana 010000, Kazakhstan; 2 Department of Political Science, Nazarbayev University, Astana 01000, Kazakhstan; augustina.alegbe@nu.edu.kz (A.O.A.); adaora.emehel@nu.edu.kz (A.N.E.); 3 WHO Country Office in Kazakhstan, Astana 010000, Kazakhstan; aubakirovab@who.int; 4 School of Medicine, Nazarbayev University, Astana 010000, Kazakhstan; yuliya.semenova@nu.edu.kz

**Keywords:** global health, health inequalities, health communication, crisis communication, health management, pandemic preparedness

## Abstract

This scoping review examines public health communication across nine Eastern European and Central Asian states—Armenia, Azerbaijan, Belarus, Kazakhstan, Kyrgyzstan, Russia, Tajikistan, Turkmenistan, and Uzbekistan—highlighting how these systems have transitioned from Soviet-era legacies to contemporary practices. Eligibility criteria included the English- and Russian-language literature published from 1998 onwards, focusing on nine post-Soviet states. Sources of evidence comprised searches in Google Scholar, ScienceDirect, SSRN, Heliyon, MEDLINE/PubMed, and official government websites. Data were charted by three independent reviewers using a standardized form, with discrepancies resolved by senior reviewers. The review identifies persistent gaps in communication during health crises, with a particular focus on the COVID-19 pandemic, where centralized and hierarchical information flows often undermine transparency and responsiveness, as well as further increased health inequalities between rural and urban health outcomes. Despite ongoing reforms, the communication dimension of healthcare systems remains underdeveloped. Findings reveal that centralized and top-down communication remains a dominant feature across the region, hindering timely dissemination of information and limiting the capacity to counter misinformation, as both misinformation and disinformation sometimes emerge from the government. Ultimately, this review contributes a critical analysis of these systematic communication failures and underscores the need to strengthen public health communication and reduce health inequalities. To do it, governments must prioritize transparency, disclose decision-making processes, and rely on evidence-based messaging to build trust. Effective crisis response requires not only government leadership but also the active engagement of the medical and patient communities, supported by civil society and independent media. This review points out the need for more inclusive, transparent, and trust-oriented communication strategies to enhance public health preparedness and resilience in nine Eastern European and Central Asian contexts.

## 1. Introduction

Over the recent years, following the COVID-19 pandemic, many governments worldwide have prioritized improving Risk Communication, Community Engagement, and Infodemic Management (RCCE-IM), analyzing the Public Health Communication (PHC) strategies employed during the crisis [1].

The evolving nature of the novel coronavirus required continuous updates and adaptable messages from governments and public health authorities (PHAs) to respond to the changing situation effectively. Public Health Communication during the pandemic is one of the core responsibilities of the government and public health agencies (PHAs), as the effectiveness of response, control measures, and readiness depends on the accurate dissemination of health information and messaging that inform and empower individuals to make informed health decisions [2]. Further, effective health communication promotes positive behavior among citizens and cultivates trust in government [3]. This need for agility was critically important in the face of rampant misinformation, often amplified on social media [4]. Pandemics often involve uncertainty, knowledge and information gaps which intensify demand for accurate and reliable health-related information [2]. The absence of trustworthy and reliable communication from government agencies can lead to public dissatisfaction and distrust, which can further hinder effective response efforts [3,4]. The pandemic thus served as a global stress test for crisis and emergency risk communication (CERC) frameworks, which emphasize transparency, credibility, and public partnership as pillars of effective response.

A significant concern for global health preparedness is the declining funding to the World Health Organization (WHO). Assessed contributions have dropped from 67% in 1971 to just 18% in 2020, limiting WHO’s capacity to prioritize proactive pandemic prevention rather than reactive measures [5]. This funding shortfall threatens the sustainability of preparedness strategies, especially in low- and middle-income countries with limited resources [5,6]. Furthermore, weak institutional frameworks and low trust in government-linked information sources undermine effective health communication [7]. These challenges are particularly acute in contexts where the institutional architecture of the state and its health system historically constrain communication practices.

### 1.1. Theoretical and Contextual Framework

This scoping review is guided by the premise that public health communication cannot be understood in isolation from the institutional structures that produce it. We draw on institutional theory, the Semashko model, and the path dependency in post-Soviet states, to analyze how deeply embedded historical legacies shape contemporary practice [8,9]. The dissolution of the Union of Soviet Socialist Republics (USSR) in 1991 created 15 independent republics, each inheriting the Semashko model of healthcare—a system characterized by extreme centralization, state ownership, and hierarchical governance [10]. Institutional theory suggests that such foundational structures create self-reinforcing paths that are resistant to change. Consequently, we argue that the public health communication challenges witnessed during the COVID-19 pandemic in these states—including top-down messaging, lack of transparency, and eroded public trust—are direct institutional outcomes, not merely operational failures. This review also engages with Crisis and Emergency Risk Communication (CERC) principles and trust-building models to evaluate the gap between established best practices and the on-the-ground realities shaped by these persistent institutional frameworks.

### 1.2. Aims and Scope of the Review

**Figure A1:**
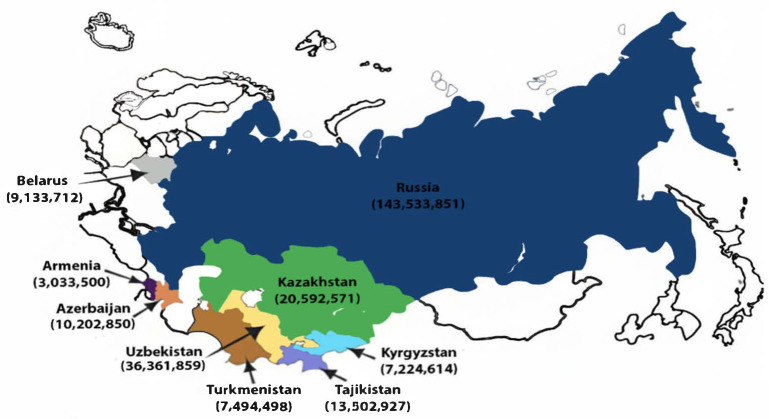
Country and Population. Countries included in the scoping review and their population as at 2024. The map was created based on the data obtained from the World Bank [145].

While the group of post-Soviet states is diverse, this review focuses on nine nations–Armenia, Azerbaijan, Belarus, Kazakhstan, Kyrgyzstan, Russia, Tajikistan, Turkmenistan, and Uzbekistan (Figure A1). This is because they share a pronounced continuity of Soviet-era institutional patterns in governance and healthcare, in contrast to Baltic or Eastern European states that have pursued deeper integration with Western structures. A scoping review methodology was selected to systematically map the breadth of available evidence, identify key concepts and gaps, and summarize findings from a diverse and interdisciplinary body of literature.

This study aims to identify the key public health communication challenges encountered by these nine post-Soviet states during the COVID-19 pandemic to understand how past legacies shape present-day strategies and approaches in public health messaging. It also aims to examine the impact of past healthcare systems and public health communication practices in these nations on their responses. By extracting insights from these aspects, this review seeks to inform and improve future health crisis strategies. The team proceeded with the review’s preparation by formulating three research questions (RQs).

- RQ1: What were the main public health communication challenges encountered by post-Soviet countries during the COVID-19 pandemic?
- RQ2: In what ways did the historical background of public health communication in post-Soviet nations shape the way they responded to COVID-19?
- RQ3: What lessons can be learned from the public health communication experiences of post-Soviet countries during COVID-19 to improve future health crisis communication?

The review offers a comprehensive overview of the key health communication challenges encountered during the COVID-19 crisis within these nations, including issues related to misinformation, public trust, and public engagement. It highlights the complex environment in which health messages are conveyed and received. Finally, the review reflects on how these insights can inform and improve future health communication strategies and practices. These insights can allow various stakeholders to develop valuable insights into culturally tailored communication strategies and underscores the importance of building and maintaining public trust during health crises.

## 2. Materials and Methods

This scoping review was conducted according to Joanna Briggs Institute methodology [11] for scoping reviews and is reported in line with the Preferred Reporting Items for Systematic Reviews and Meta-Analyses extension for Scoping reviews (PRISMA-ScR) guidelines [10]. A completed PRISMA-ScR checklist is provided in Appendix A (Table A1).

### 2.1. Protocol and Eligibility Criteria

A priori protocol was not developed or registered for this scoping review. Although not preregistered, the methodology was designed to adhere to established scoping review standards to ensure systematicity and transparency. The review involves the analysis of academic publications dedicated to the discussions on transformation of public health communications in post-Soviet states since the late 1990s. The review is focusing on nine of fifteen post-Soviet states–Russia, Belarus, Kazakhstan, Kyrgyzstan, Tajikistan, Turkmenistan, Uzbekistan, Armenia and Azerbaijan. The six states–Estonia, Latvia, Lithuania, Moldova, Ukraine, and Georgia–are excluded. This is because of their emphasis on integration into European and Western institutions, such as the EU and NATO, which requires adopting governance standards focused on transparency and public participation. As a result, their approach to public health governance differs significantly, aligning more with Western European models than with the centralized, top-down systems that characterize the nine post-Soviet states included in this review.

Inclusion and exclusion criteria were applied to the literature as follows:

Inclusion criteria:
  - Population/context: studies focusing on one or more of the nine defined post-Soviet states.
  - Concept: studies discussing public health communication, health systems, crisis communication, risk communication, or related challenges (e.g., misinformation, public trust, infodemic).
  - Timeframe: Literature published from 1998.
  - Types of Sources: Academic publications (original research, reviews), government reports, policy documents, and analyses from international organizations (e.g., WHO, UNICEF, World Bank).
  - Languages: Sources published in English or Russian.
Exclusion criteria:
  - Studies focused exclusively on the six excluded post-Soviet states (Estonia, Latvia, Lithuania, Moldova, Ukraine, Georgia).
  - Studies published before 1998.
  - Sources not available in English or Russian.
  - News articles, opinion pieces (unless published in an academic or official report format), and non-peer-reviewed blog posts.
  - Studies where the full text could not be retrieved.

### 2.2. Information Sources and Search Strategies

A comprehensive search strategy was designed to identify both published and gray literature. The final search was executed on 27 September 2025. The primary database used for our search was Google Scholar, chosen for its wide coverage of interdisciplinary academic literature. We also sourced for articles on ScienceDirect, SSRN, Heliyon, and MEDLINE/PubMed. Government websites such as the Ministry of Healthcare of Kazakhstan and the Ministry of Health of the Republic of Uzbekistan were also consulted to supplement academic sources, particularly given the limited number of publications on health communication in post-Soviet countries. To identify additional sources, we manually searched the websites of relevant government health ministries and international organizations (e.g., WHO, World Bank). Furthermore, the reference list of key articles were screened for eligible studies.

The search strategy used a combination of keywords and Boolean operators (AND, OR) related to the country names, key concepts, and context. The search strategy was first developed for Google Scholar and then adapted for the other databases. The full electronic search strategy for Google Scholar is provided in Appendix A (Table A2) to ensure reproducibility.

### 2.3. Selection of Sources of Evidence

**Figure A2:**
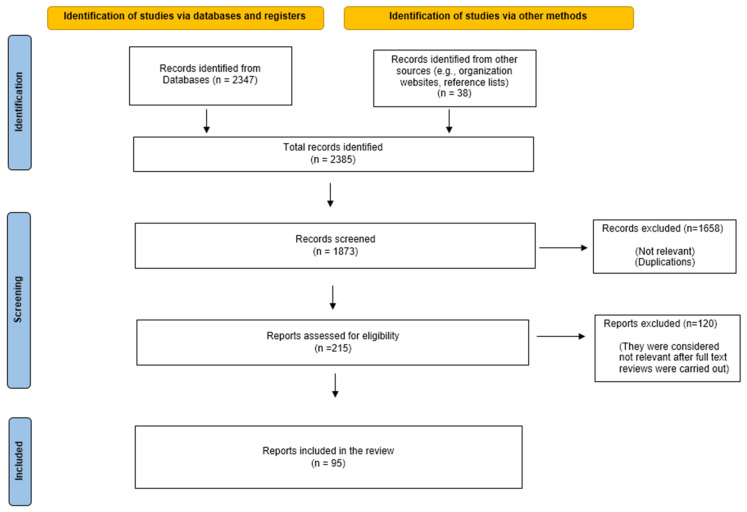
PRISMA Flow Diagram.

The source selection process, detailed in the PRISMA-ScR flow diagram (Figure A2), involved a systematic screening approach. After search results were collated and duplicates removed, three reviewers (A.M., A.A. and A.E.) independently screened records in two phases. First, titles and abstracts were assessed against the eligibility criteria. Subsequently, the full texts of potentially relevant sources were retrieved and evaluated. Any disagreements at either stage were resolved through consensus or by consultation with a senior reviewer (L.L. or Y.S.).

### 2.4. Data Charting Process and Data Items

Data from the included sources were charted using a standardized data charting form, which was developed by the research team and piloted on five random sources to ensure consistency. The charting process was conducted independently by three reviewers (A.M., A.O.A. and A.N.E.), with periodic meetings to compare results and ensure uniformity. The following data items were systematically extracted from each included source:

- Bibliographic details: Author(s), publication, title, source.
- Context: Country or countries of focus.
- Key findings: Relevant data pertaining to research questions, including:
  - Historical background of the health system
  - Regional healthcare inequalities
  - Health communication practices
  - Identified challenges during the COVID-19 pandemic (misinformation, public trust, politicization)

### 2.5. Critical Appraisal of Individual Source of Evidence

Consistent with the purpose of the scoping review to map the available literature rather than to weigh the evidence from individual studies, a formal critical appraisal (risk of bias assessment) of the included sources was not conducted.

### 2.6. Data Synthesis

The extracted data were synthesized using an integrated qualitative approach combining qualitative content analysis, thematic analysis, and narrative synthesis.

First, qualitative content analysis [12] was used to systematically code the charted textual data, categorizing key concepts and insights relevant to the research questions.

Next, we applied the principles of reflexive thematic analysis [13] to the coded data. This involved an active, iterative process of reviewing codes, generating candidate themes, refining them against the full dataset, and defining and naming the final thematic framework.

Finally, a narrative synthesis [14] was conducted. The findings were organized according to the final thematic framework, and a narrative summary was developed to interpret and relate the evidence across sources, explaining connections and contradictions within the literature. This synthesis is presented narratively and supported with tables and figures to provide a comprehensive descriptive overview of public health communication in the nine post-Soviet countries.

To ensure methodological rigor, we employed a differentiated analysis strategy for source types. Academic literature was analyzed to identify thematic challenges, critical evaluations of response efficacy, and public sentiment. In contrast, government documents and policy reports were extracted solely for contextual data—specifically to map the timeline of regulatory measures, official communication channels used, and stated policy goals. We were careful not to conflate official policy statements with empirical evidence of their implementation; for instance, claims regarding “transparency” or “trust” are derived exclusively from empirical academic studies and independent international assessments, rather than government self-reporting.

### 2.7. Key Concepts and Definitions

For the purpose of this review, we distinguish between two related but distinct concepts. We define public health communication (PHC) broadly as the strategic use of communication to inform and influence individual and community decisions that enhance health [15]. This encompasses routine health promotion, disease prevention campaigns, and long-term health literacy efforts. In contrast, we define risk communication and community engagement (RCCE) as a specific component of health emergency preparedness and response. It refers to the real time exchange of information, advice, and opinions between experts or officials and the public facing a threat to their survival, health or well-being [16].

## 3. Results

### 3.1. Selection and Characteristics of Sources of Evidence

The systematic search and selection process are detailed in the PRISMA-ScR flow diagram (Figure A2). The initial search identified 2385 records from databases and other sources. After removing duplicates, 1873 records underwent title and abstract screening. Of these, 215 full-text sources were assessed eligibility. A total of 95 sources met the inclusion criteria and were included in the final scoping review. The primary reasons for exclusion at the full-text stage were: not focusing on the nine specified countries (*n* = 67), not addressing public health communication (*n* = 42), and other (*n* = 11).

The characteristics of the 95 included sources are summarized in Table A3. The evidence base comprised a diverse range of document types, including peer-reviewed journal articles (*n* = 51), working papers, block posts or conference papers (*n* = 6), and books or book chapters (*n* = 11). Additionally, it included reports and reviews from international organizations like the World Health Organization, UNICEF, and World Bank (*n* = 22), and press-releases from official websites of governmental agencies (*n* = 5). Geographically, the sources covered all nine countries, with many addressing multiple countries or the region as the whole. The publication dates ranged from 1998 to 2025, with a significant concentration (around 50%) from the COVID-19 pandemic period (2020–2023).

### 3.2. Thematic Synthesis of Results

The findings of the scoping review were grouped into major recurring themes and patterns identified during the article search process. Specifically, the thematic structure is organized around (1) the continued influence of Soviet-era governance and communication legacies, (2) the public health communication challenges observed by the states under consideration during COVID-19, and (3) the lessons and the recommendations the scholars offer for future improvement in the field.

The following sub-section provides the historical background and structure of healthcare systems during the Soviet and post-Soviet eras. This context provides useful information in understanding how institutional legacies have shaped contemporary public health communication practices. We begin by analyzing the legacy of Soviet-era health systems that determined the creation of highly centralized and hierarchical structures in the post-Soviet period. This, in turn, shaped the development of centralized public health communication practices.

#### 3.2.1. Background of Healthcare Systems During and Post-Soviet Era

During the Union of Soviet Socialist Republics (USSR), most countries adopted the Semashko healthcare systems [17]. The Semashko model is a centralized, state-run healthcare system developed in the Soviet Union under People’s Commissar for Health Nikolai Semashko in the 1920s. The system is characterized by universal access to free medical care, financing through general taxation, state ownership of healthcare facilities, and a hierarchical administrative structure based on territorial units (republic-oblast-rayon) [17]. At its core, the model prioritized the control and prevention of diseases, while emphasizing accessible basic primary care for all citizens. Although it was considered a “coherent and cost-effective model” [18] (p. 2) that significantly improved health outcomes in the USSR, this model was characterized by centralized and hierarchical government management [18].

Critics argue that declining quality in services stemmed from underfunding and lack of resources, leading to diminishing returns over time [17,18]. The critique highlights the inherent flaws in the system that, despite its initial success in controlling diseases and providing accessible primary care, struggled to maintain quality due to financial constraints and resource limitations. As a result, the Semashko model faced challenges and influenced overall public health outcomes.

After the fall of the USSR, the Semashko healthcare model continued to influence healthcare organization and governance in the post-Soviet states [18,19]. During the healthcare reforms, many post-Soviet nations faced considerable challenges. These challenges included economic crises caused by hyperinflation, shifts from state-controlled to market-controlled economies, governance weakness arising from these transitions, persistent corruption issues rooted in the Soviet Union, low wages for medical professionals, and protests from healthcare workers [12,17]. These challenges have often resulted in fragmented healthcare systems that struggle to improve and provide quality care.

During the healthcare transition from the Semashko system to the social health insurance scheme in the Central Asia states, Armenia, and Azerbaijan, reforms focused on strengthening primary care to improve efficiency and access continue to face challenges, including inadequate governance, economic difficulties, accountability, and transparency [20]. Armenia and Azerbaijan encountered further obstacles stemming from an ethnic and disputed territorial conflict between Armenia and Azerbaijan over the region of Nagorno-Karabakh, which impeded the progress of reforms. Although there has been some progress since 2020, persistent economic constraints, underinvestment, and weak institutions continue to impede health system advancements throughout these regions [21]. Nonetheless, Belarus and Russia implemented an incremental reform strategy, utilizing pilot projects to evaluate models prior to nationwide implementation of successful components, emphasizing sustained access rather than swift systemic transformation [22].

**Figure A3:**
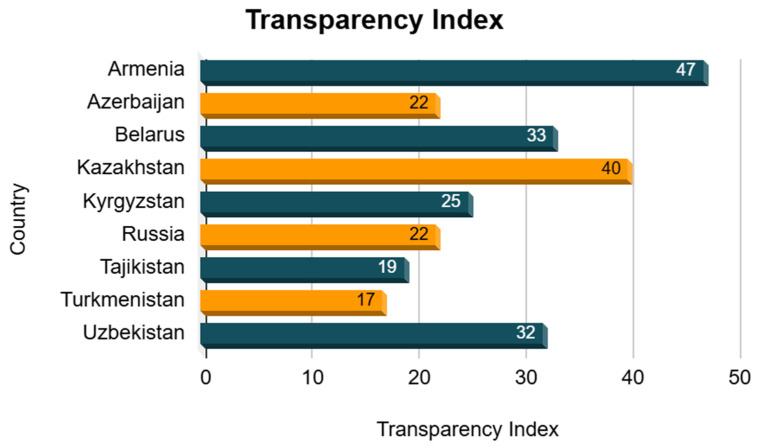
Transparency Index. This chart was created with data obtained from Transparency International for 2024 [146]. Classification if from 0–100; 0-highly corrupt, 100-very clean.

Transparency Index (Figure A3) illustrates the varying levels of perceived public sector transparency across the studied post-Soviet states, providing context for their differing reform trajectories. The path of health system changes in these states illustrates a complex interaction between economic difficulties, institutional weakness, and the strategic decision-making processes underlying healthcare reform. Despite facing significant hurdles such as persistent economic constraints and institutional weaknesses, some countries like Armenia (Transparency Index score: 47) and Kazakhstan (Transparency Index score: 40) have adopted an incremental reform strategy that prioritizes sustained access to healthcare over immediate, sweeping changes.

In contrast, persistent economic limitations and corruption as reflected in lower scores for Russia (Transparency Index score: 22), Tajikistan (Transparency Index score: 19), and Turkmenistan (Transparency Index score: 17) hinder progress. The path forward for these nations requires them to navigate a range of obstacles using a balanced approach. This approach must address both immediate healthcare needs and the long-term sustainability of healthcare delivery.

This pattern underscores the enduring Soviet legacy of hierarchical governance in healthcare.

#### 3.2.2. Centralized Hierarchical Healthcare

The state owned and managed all facilities in the highly hierarchical and centralized healthcare system of the former USSR [23]. Following the dissolution of the USSR in the 1990s, various post-Soviet nations initiated reforms to reduce state involvement in healthcare delivery. These reforms included significant structural and regulatory changes, as well as the privatization of state-owned healthcare facilities. However, these initiatives faced constraints due to inadequate funding, poor facilities, and corruption [23,24].

As a result, most post-Soviet nations continued to operate centralized healthcare systems inherited from the USSR, focusing on providing basic primary healthcare access while facing significant obstacles. Russia, Belarus, and Armenia have centralized hierarchical governance structures with limited reforms, frequently obstructed by financial constraints and disparities between urban and rural regions [22,25,26]. Kazakhstan and Azerbaijan have commenced decentralization initiatives by establishing agencies and reallocating responsibilities [23,27,28,29], yet significant central control remains noticeable. Uzbekistan and Tajikistan continue to operate healthcare systems dominated by government control, with minimal involvement from civil society and the private sector [30,31,32].

Despite healthcare reforms intended to enhance efficiency and decentralization, many countries still encounter structural limitations, unequal service delivery between urban and rural areas, and an ongoing dependence on the centralized planning inherited from their Soviet legacy [33,34,35]. These challenges hinder the potential for genuine progress in health outcomes and equity. As a result, stakeholders are increasingly advocating for a more integrated approach that emphasizes community engagement and encourages investment in local health initiatives [27,29].

Persistent structural weaknesses in governance and funding have, in turn, led to significant financial constraints for these healthcare systems.

#### 3.2.3. Financial Constraints of Healthcare Systems

Post-Soviet healthcare systems primarily rely on government funding, a legacy of the Soviet era, but the specific funding models and recent trends reveal significant challenges. The financing of Russia’s healthcare system is predominantly sourced from federal and regional budgets; however, economic limitations hinder resource distribution and reform efforts [25]. Similarly, Belarus depends significantly on centralized government funding, while regional authorities manage resource allocation, though, disparities persist due to varying fiscal capacities [22].

In Central Asia, approaches have varied. Kyrgyzstan pioneered health financing reforms by introducing mandatory health insurance in 1996 and capitation payments for primary care, later experimenting with performance-based funding (2018–2021) [29,36]. Uzbekistan followed with capitation payments, adjusted for age, gender, and regional budgets [37]. Kazakhstan undertook broader reforms from 2009, harmonizing tariffs and combining capitation with pay-for-performance indicators [38]. Tajikistan introduced partial capitation in 2010 and formally expanded it in 2019, but in practice financing remains input-based and fragmented, leading to inefficiency [31].

Elsewhere in the region, Armenia primarily relies on government allocations, resulting in restricted private or external funding, which contributes to resource limitations and inefficiencies [39]. Azerbaijan and Kazakhstan have sought to diversify their financing sources via regional agencies and insurance schemes, yet government budgets continue to serve as the principal mechanism for funding [40].

As illustrated in Table A4, state healthcare budgets primarily fund the healthcare systems of post-Soviet countries, with minimal alternative or supplementary funding sources available. The data reveals a concerning trend: while seven other post-Soviet nations have reduced their health budgets, only Belarus and Uzbekistan were increasing their health expenditure. Overall, underfunding and underinvestment remain prevalent across post-Soviet countries, limiting the quality, accessibility, and resilience of their healthcare systems. These financial constraints continue to hinder effective public health preparedness and crisis response in the region today.

These systematic and structural financial challenges manifest most visibly in stark inequalities in healthcare access.

#### 3.2.4. Regional Healthcare Inequality Between Rural and Urban

Post-Soviet nations show substantial disparities in healthcare access and quality between rural and urban regions [18]. Rural populations frequently have access only to basic services, requiring long travel distances for serious conditions such as cancer or surgical procedures [18]. Additionally, rural regions struggle to attract and retain medical personnel. For instance, only 17% of Kazakh doctors serve rural populations [41]. As a result, healthcare professional shortages are significant, particularly as urban areas focus on advanced treatments. Additionally, hospitals in rural areas face a lack of infrastructure, a lack of medical supplies, and a shortage of specialists, all of which contribute to disparities, especially in rural clinics that lack essential technologies [41]. Semenova et al. [18] point out that the migration of medical workers to more affluent neighboring regions and overseas employment leads to a shortage of expertise in rural areas.

Moreover, regional disparities in funding [8,26,29] and inadequate government standards [42] exist. These issues include inadequate transport infrastructure and limited access to regional medical centers [42]. The lack of adequate standards and infrastructure creates significant inequalities, leading to reduced accessibility, lower quality of healthcare, and insufficient capacity to address local healthcare needs in rural communities [41]. In Armenia, Quliyeva and Huseynov [42] highlight that the lack of national standards or planning guidelines has resulted in significant disparities in the availability of medical equipment and the training of healthcare staff across regions. Furthermore, there is a shortage of health specialists in rural areas due to the absence of incentives for physicians to relocate there. In Belarus, urban areas rely on polyclinics with specialists, while rural areas depend on family doctors and outpatient clinics, which are often understaffing issues [8]. The increased strain on healthcare workers not only affects their well-being, but also diminishes the quality of care they can offer to patients.

Additionally, shared salaries for vacant posts discourage recruitment efforts, and the lack of consultation privacy undermines patient–doctor communication [8]. Azerbaijan also experiences a considerable disparity in the quality of medical services between rural and urban areas. Quliyeva and Huseynov [42] attribute this phenomenon primarily to inadequate transport infrastructure, which limits access to regional medical centers. However, in the past decade, the number of health workers per capita has declined, further accentuating disparities between urban and rural settings. Rural regions face persistent staffing and infrastructure shortages and struggle to attract and retain physicians, largely due to low salaries and challenges associated with replacing retiring doctors 22. This urban bias exacerbates access disparities, leading to longer patient wait times and diminishing the quality of care in rural regions [26].

Moreover, this situation intensifies health disparities and places significant pressure on the remaining healthcare professionals, who frequently face overwork, underpayment, and inadequate resources [18]. This disparity results in unequal healthcare services. Urban areas benefit from advanced medical technology and well-trained professionals, while rural areas face challenges with outdated equipment and a shortage of medical professionals. As a result, patients in under-resourced regions encounter challenges in receiving appropriate medical care, and the cycle of health inequality continues, further worsening the challenges encountered by rural communities in the post-Soviet nations.

These deeply integrated structural features of post-Soviet healthcare systems directly shape how health information is communicated to the public. Following the historical overview of health systems and the challenges faced during and post-Soviet era, the subsequent sections present the identified themes as a thematic synthesis of the literature. It expands on the three main themes identified earlier: the continued influence of Soviet-era governance and communication legacies, the public health communication challenges observed by the states under consideration during COVID-19, and the lessons learned and the recommendations the scholars offer for future improvement in the field.

#### 3.2.5. Health Communication: Soviet Legacy to Contemporary Practice

The centralized political culture of the USSR heavily shaped public information flows and media systems [43]. During the USSR period Freedman et al. [43] note that journalists, despite being technically skilled, worked under strict ideological constraints. Their primary role was to highlight the achievements of the Communist Party and the state through propaganda, while minimizing or overlooking negative or controversial issues to maintain a positive and favorable image of the government. Endaltseva [19] highlights that a lingering Soviet legacy is the interpretive reporting style, where journalists’ opinions replace objective coverage. In the post-Soviet context, this interpretive approach has been shaped by authoritarian regimes, resulting in reporting aimed at appeasing political power rather than informing the public [19].

Such practices have consequently weakened the quality of information and diminished public confidence in media systems [43]. The erosion of trust had significant consequences for public engagement and accountability, making it difficult for citizens to differentiate between truth and propaganda. As a result, the media landscape became increasingly polarized, complicating the relationship between the public and those in power. Furthermore, public health communication was either nonexistent or minimal during the USSR period [19]. This lack of effective public health communication not only limited citizens’ access to essential health information and policies but also worsened public health crises, allowing misinformation to spread unchecked.

After the collapse of the USSR, communication remained predominantly centralized, mirroring the Semashko model’s top-down governance [19]. This centralization is not only geographic but also institutional, as communication often targets key stakeholders within the health and financing sectors rather than the general public. This hierarchical structure limits responsiveness to local needs, with information flowing vertically and bureaucratically rather than horizontally and adaptively [19]. There has been little progress in developing standardized professional health communication practices for the post-Soviet countries.

In Russia, public health communication serves as a tool to navigate and compensate for systemic inconsistencies [19]. The government in Belarus recognizes the need for patient education materials. However, official medical portals lack essential guidance on where citizens can seek advice and assistance [8,44]. Similarly, in Kazakhstan, public health communication has rarely been prioritized and suffers from a lack of dedicated funding allocation or government support [45,46]. The implementation of major health policy changes often occurred without adequate explanation to the public or frontline health workers, which contributed to misinformation, confusion, and a lack of public trust.

More broadly in Central Asian states, public health communication campaigns have often relied on support from international organizations like UNICEF and WHO [47]. Key interventions included advocacy efforts directed at ministers, parliament members, regional governors, and community leaders to secure cross-sector buy-in.

Additionally, outreach to mass media was conducted to distribute promotional materials to rural families, along with social mobilization that engaged other ministries and agencies [47]. Strategic communication reforms, digital transformations, and international partnerships are increasingly shaping contemporary health communication in Armenia [48]. Before 2007, Azerbaijan’s health communication suffered from weak coordination, limited resources, and inconsistent messaging. The creation of the Public Health and Reform Centre (PHRC) and its Department of Health Communication and Public Relations (DHCPR) improved oversight, streamlined activities, and, with international support, led to a stronger national health communication strategy [49].

The practice of health communication is influenced by a wide and often fragmented set of institutional actors.

#### 3.2.6. Health Communication and Communicators Across the Post-Soviet Nations

Most post-Soviet countries recognize the Ministry of Health as a central actor in public health communication, responsible for disseminating information about health policies, initiatives, and campaigns. Their role is crucial in ensuring that the population receives accurate and timely health information. Yet, public health communication involves a wide range of stakeholders, each pursuing their own goals with varying degrees of coordination [19,41]. The complexity reveals significant challenges, including unclear roles and identities among public health communicators [41,49], which hinder the development of effective communication strategies and diminish community engagement. As a result, misunderstandings and misinformation often spread, reducing the resonance and effectiveness of health messages.

Endaltseva [19] identifies key actors involved in health communication, such as government agencies promoting policies, patient associations advocating for patients’ rights, pharmaceutical companies targeting consumers, NGOs and charities, insurance companies, and educational and research institutions. The diversity of these actors underscores the fragmented nature of health communication systems in post-Soviet nations. Such fragmentation can produce mixed messages, misinformation, and public confusion, ultimately undermining trust and making it difficult for individuals to make informed health decisions. Endaltseva [19] emphasizes that this lack of cohesion weakens health messaging, lowers public confidence, and hampers overall health outcomes. She advocates for improved collaboration and alignment among stakeholders to enhance clarity, accuracy, and accessibility of health information, which is crucial for better public health results. Traditional and social media both play a crucial role in increasing public awareness and educating the citizens about health issues in post-soviet countries [50,51]. Antonova [51] notes the importance of various media platforms as tools for disseminating crucial health information and policies, as well as fostering informed health decision-making among the populace.

Despite this potential, the overall effectiveness of media communication is often limited by insufficient government engagement in actively disseminating health information [52,53]. Public understanding and compliance with health messages tend to remain low without strategic and sustained governmental support [52]. Although some scholars emphasize the individual responsibility for seeking out and interpreting health information [53], evidence indicates that personal efforts alone are insufficient to bridge the communication gap. Here, they stress that strong government involvement through clear messaging, coordinated campaigns and backing media initiatives are essential to convert media outreach into meaningful public understanding and behavioral change [52]. Effective government participation is critical to ensuring that well-intentioned health initiatives have their intended impact on community health outcomes.

In Russia, a grassroots advocacy movement emerged in the 1990s as a response to the lack of government support in health communication. Over time, these movements serve as mediators, helping to bridge the communication gaps between official messaging, healthcare providers and the public [19]. They play a vital role in fostering dialog and understanding among various stakeholders, ensuring that public health messages are effectively conveyed and understood to improve health outcomes. Nevertheless, the encounter substantial obstacles, particularly the pervasive influence of aggressive pharmaceutical marketing that has increasingly dominated health messaging in the media [52].

This dominance makes it difficult for grassroots advocates to ensure the dissemination of clear and accurate health information. Health messages driven by commercial interests have also led to widespread self-treatment practices and patients’ reluctance in seeking professional care. Such trends complicate the role of health mediators and as such, pose risks to patient safety and well-being. As individuals continually rely on self-diagnosis, the potential for misinformed decisions rises, highlighting the need for unbiased and accessible health communication. These issues are further compounded by the Russian state’s insufficient public health promotion efforts and the absence of targeted health communication campaigns promoting healthy lifestyles [52].

In Belarus, the health system is highly centralized, with the Ministry of Health setting priorities and policies that cascade down through district, interdistrict and regional levels. Public health communication follows a similar top-down approach, with the government largely controlled at the national level before being disseminated locally [54]. The system retains largely Soviet-era characteristics, marked by centralized hierarchical practices. Civil society and the population have limited involvement in shaping health priorities or policies. Patient organizations are few, mainly focused on specific disease groups, and some maintain links with professional specialist associations [8]. Healthcare professionals looked upon as the primary, often the sole, responsible actors for health, which results in communication that is directive and top-down, emphasizing compliance over dialog. Consequently, patient adherence to treatment remains weak, reflecting the limitations of this paternalistic communication style [8].

In Kazakhstan, the Ministry of Health is primarily responsible for public health communication, with the Department of Public Relations (DPR) managing its implementation. At the regional level, Health Departments collaborate with local health workers to ensure that messages are both culturally relevant and consistent, thereby improving their reach to diverse communities [55]. Iskakov [56] emphasizes the crucial role of healthcare leaders in engaging media to foster public trust and improve the healthcare system’s reputation. The article [56] highlights the importance of proactive communication strategies in shaping public perceptions and overcoming the legacy of Soviet influence in Central Asia, arguing that effective communication can build trust and strengthen relationships between health authorities and the public. However, Iskakov [56] notes a significant gap in strategic communication, with no clear overarching strategy to guide outreach efforts. This shortcoming often results in misunderstandings and mistrust, undermining health initiatives. Furthermore, the limited use of digital platforms for outreach hinders access to vital health information and reduces opportunities for timely engagement with diverse audiences, restricting the ability to address public concerns effectively.

In Uzbekistan, the management of health communication is through a collaborative framework involving government agencies, international organizations, non-governmental groups, and healthcare practitioners. The Ministry of Public Health serves as the primary authority, responsible for developing policies, organizing initiatives, and overseeing communication efforts via its website, press service, and communication centers. The Service for Sanitary Epidemiological Welfare and Public Health leads campaigns, community engagement, and misinformation control, especially during emergencies, with support from the Public Health Emergency Operations Center. International agencies like WHO and UNICEF provide technical assistance, support vaccination drives, and share best practices. Key communicators include healthcare professionals, government officials, media specialists, and community leaders who convey health messages and build public trust.

Uzbekistan emphasizes digital health projects involving experts in e-health, telemedicine, and social media to combat disinformation and enhance outreach, particularly during the COVID-19 pandemic [57,58]. This comprehensive approach aims to improve health literacy, foster trust, and strengthen system responsiveness nationwide. However, the country’s health communication efforts face challenges such as fragmented coordination, unclear institutional roles, limited resources and technical capacity, inconsistent messaging, low public trust, digital divides, weak monitoring and evaluation, reliance on short-term donor projects, bureaucratic delays, and limited crisis communication preparedness [56,59]. These issues disproportionately affect rural and vulnerable populations, hindering equitable health outcomes [60,61,62].

In Kyrgyzstan, the Ministry of Health oversees health communication initiatives, supported by specialized entities such as the Republican Center for Health Promotion and Mass Communication. The ministry develops national health policies, including the ‘Healthy Person–Prosperous Country’ plan (2019–2030), which sets communication priorities. International organizations like WHO, UNICEF, USAID, and GIZ provide technical and financial support, especially in crises and digital health projects. Healthcare professionals, including primary care providers and mobile vaccination teams, are vital in informing and engaging remote populations. Health facilities and the Mandatory Health Insurance Fund also serve as communication channels. Civil society organizations and media both–traditional and social–play crucial roles in disseminating health messages, promoting healthy lifestyles, and counteracting misinformation [47]. Kyrgyzstan’s digital infrastructure, including electronic health records, telemedicine, and mobile apps, supports its strategic goal to inform, engage, and empower citizens [36,63].

The Republican Centre on Health Promotion and Communication, under the Ministry of Health, is the main agency responsible for health promotion and information dissemination, implementing initiatives like education campaigns and community outreach. Funded by the national budget, the Centre’s responsibilities expanded in 2019 to include direct population engagement [36]. Despite these efforts, there is limited research on the effectiveness of Kyrgyzstan’s health communication strategies, highlighting a significant gap in understanding their impact on public health outcomes. Addressing this knowledge gap could improve current initiatives and inform future policies.

In Tajikistan, the Ministry of Health and Social Protection of the Population is primarily responsible for health communication, overseeing public health messaging, disease prevention campaigns, and health education initiatives [64]. Regional and local health authorities, alongside international partners such as the World Health Organization, also contribute to these efforts. Community members, especially in highland and remote areas, serve as health communicators, engaging as public activists or members of self-help groups to address infrastructure limitations in rural regions [65].

However, he notes that reliance on community members may lead to inconsistencies in information, as not all individuals have adequate training or understanding of health issues [65]. He argues that this approach can also inadvertently create disparities in access to accurate health information, particularly when engagement levels vary among community groups [65].

In Armenia, the Ministry of Health leads the development and implementation of health policies, including communication strategies. Regional authorities coordinate local efforts, while the National Emergency Services are active during public health emergencies. NGOs such as the Armenian Association of Digital Health (AADH) and international partners like WHO, UNICEF, and USAID support health communication initiatives. Healthcare professionals, including doctors and public health officials, serve as direct sources of information, sharing vital health updates with the public and patients. Educational institutions, particularly Yerevan State Medical University, contribute to health education through digital platforms, telemedicine, media outlets, and government-sponsored eHealth systems. Armenia’s health communication efforts involve multiple actors—government agencies, NGOs, international organizations, healthcare professionals, and digital channels.

However, the focus is primarily on emergencies and outbreaks, with less emphasis on routine health messaging or disseminating scientific information. Despite having trained spokespeople in public relations departments, there is a recognized need for more proactive community engagement to strengthen risk communication and build public trust. While internal and vertical communication within ministries functions relatively well, previous simulation exercises have revealed weaknesses in inter-regional and interdepartmental (horizontal) communication [66].

In Azerbaijan, the Ministry of Health, its subsidiary the Public Health and Reforms Center (PHRC), and international organizations such as WHO and UNICEF manage health communication. The Ministry is responsible for policy-making, while the PHRC, established in 2006, focuses on developing and implementing health programs, including communication strategies. WHO and UNICEF support Azerbaijan by promoting public awareness, creating health campaigns, and providing accessible health materials, especially targeting children and parents through social media and digital platforms.

The Azerbaijan Health Communication Association (AHCA), a non-governmental organization, brings together experts in public relations, research, and communications to design campaigns and influence policy change. Key communicators include government officials from the Ministry and PHRC, international teams from WHO and UNICEF, AHCA specialists, and various NGOs. They utilize platforms such as digital media, community outreach, and mass campaigns to disseminate health information and foster public engagement. However, Azerbaijan faces significant challenges in health communication, including fragmented coordination and unclear roles, limited resources and capacity, inconsistent messaging, low public trust and misinformation, digital access gaps—particularly in rural areas—and weak monitoring and evaluation systems. Bureaucratic delays, donor-driven short-term initiatives, and inadequate crisis communication preparedness—especially affecting vulnerable rural populations—also hinder efforts [1,67,68,69].

Across post-Soviet states, similar issues persist, including limited awareness of health communication’s importance, scarce research on effective strategies, and unclear roles for communicators. Weak institutional frameworks, fragmented actors, and poor coordination lead to inconsistent messaging, public confusion, and diminished trust. These constraints obstruct the development of culturally appropriate campaigns, weaken community engagement, and enable misinformation, ultimately limiting access to vital health knowledge and perpetuating inequalities in health outcomes among vulnerable populations if not addressed swiftly.

The weaknesses of institutional framework for health communication were laid bare and intensified during the COVID-19 crisis.

#### 3.2.7. Crisis Communication During the Pandemic

Crisis communication refers to communication during an outbreak when people need to know exactly what to do if they are affected and how to protect themselves and others [67]. The COVID-19 pandemic prompted notable shifts in health communication across post-Soviet countries. During this period, there was a notable increase in the use of digital platforms and social media to reach broader audiences. Governments aimed to improve transparency and public engagement by providing daily updates, launching virtual awareness campaigns, and offering online consultations in attempts to improve transparency and to combat misinformation [70]. International cooperation and guidance were also leveraged to unify messaging and build public trust during an evolving health emergency [47]. These adaptations reflected responses to crisis conditions and highlighted the growing importance of digital tools for public health communication. However, despite increased digital adaptations, efforts did not uniformly translate into better public-health outcomes. While digital tools expanded reach, factors like misinformation, digital divides, and mistrust hampered the effectiveness of virtual campaigns, limiting their impact on behavior change and disease control [33,71].

**Figure A4:**
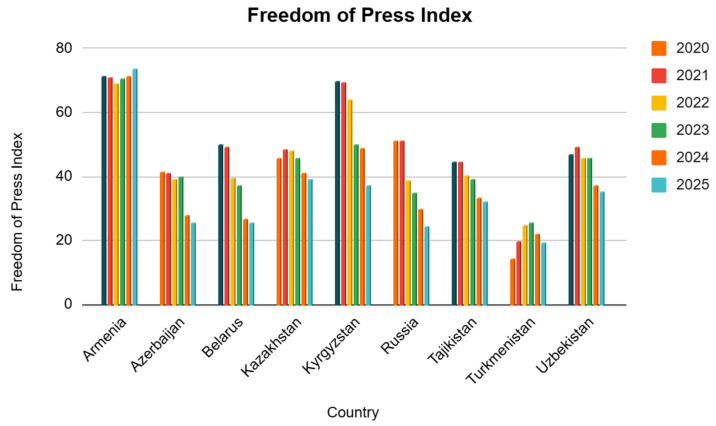
Freedom of Press Index. This chart was created with data gotten from Reporters without borders [147]. Classification: 85–100 (Good), 70–85 (Satisfactory), 55–70 (Problematic), 50–55 (Difficult), 0–40 (Very serious).

Across the region, constraints on press freedom and information access hampered transparent reporting (Figure A4). In Russia, this shift was marked by a reformatting of communication styles driven by increased demand for trust and remote interactions, but effectiveness remained limited due to legacy issues like government control over information and restrictions placed on healthcare workers [72]. Science and health journalists in Russia faced editorial restrictions, limited source access, and widespread self-censorship [73]. In Belarus, insufficient and inconsistent communication efforts, including limited press briefings and a lack of official messages, led to public confusion, misinformation, and mistrust, exacerbating pandemic challenges [74].

Kazakhstan employed a multi-platform approach, utilizing official websites, social media, and media outlets to disseminate health information [45,46]. While perceived as trustworthy, these channels often failed to reach all demographics effectively due to bureaucratic language and lack of tailored messaging, resulting in disparities in engagement. Effectiveness varied by age, occupation, and urban–rural residence [46]. Although official websites were often perceived as trustworthy, one-size-fits-all messaging and bureaucratic language limited reach—30–45-year-olds reported higher satisfaction, while younger and older groups were less satisfied—and sectoral differences shaped channel preferences [75,76]. Kazakh journalists also reported difficulties obtaining timely, comprehensive information from state bodies [77,78,79,80]. Kazakhstan, Uzbekistan, and Kyrgyzstan combined strict containment measures with broad public information campaigns, and targeted personalized appeals to citizens, reflecting authorities’ confidence in their legitimacy and an emphasis on transparency [81].

Kazakhstan and Uzbekistan, despite recording high case numbers, mobilized domestic reserves, reallocated budgets, and secured external loans to finance responses such as importing ventilators, building mobile hospitals, and carrying out large-scale public disinfection spaces. Kyrgyz authorities also demonstrated effective public information efforts. However, strategic missteps in the area of socio-economic development exposed the sector’s inability to provide broader support for pandemic response efforts [81].

By contrast, Tajikistan and Turkmenistan adopted more restrictive information postures, focusing primarily on anti-coronavirus measures while constraining public access to infection data—an approach that widened the gap between authorities and society [81]. Tajikistan authorities delayed official acknowledgement of infections, a hesitation attributed to concerns about losing control and the political context of imminent presidential elections [81]. In Turkmenistan, official channels denied or minimized domestic cases and reported that preventive measures and medical supplies were adequate. Independent and opposition media, however, described severe shortages and dire conditions for health workers, who reportedly purchased personal protective equipment themselves [81].

Similar limitations on public reporting emerged elsewhere in post-Soviet states. In Armenia, COVID-related communications were tightly centralized under the Commandant’s Office, with both media and private citizens permitted to publish only information drawn from official sources restricting independent investigation and fact-checking [82]. Low public trust in Azerbaijan impeded compliance with public health measures: the Ministry of Health relied on official websites and social media to promote mask, distancing, and hygiene rules, but persistent distrust limited effectiveness [83,84]. To address misinformation, WHO delivered training to journalists, NGOs, social media influencers, and local health representatives to build capacity for identifying and countering false information; the effectiveness of these sessions warrants further evaluation 22.

Taken together, these patterns suggest that both the content and openness of official communications–and the degree of trust they engender–were decisive factors shaping public understanding and compliance during the pandemic.

These pandemic-era challenges can be traced back to a more fundamental problem: the lack of a robust institutional framework for communication.

#### 3.2.8. Lack of Institutional Communication Framework

The institutional framework governing public health communication in post-Soviet states is often characterized by systemic gaps, leading to communication delays and inconsistencies. In Kazakhstan, the Ministry of Healthcare is the central authority responsible for public health, with its activities regularly disseminated through the Public Relations Department (PRD), which manages press conferences, media monitoring, and the Ministry’s online presence [85]. However, the PRD frequently faces staffing shortages, which limit its capacity to manage communications effectively due to overwhelm and resource constraints [45]. The Ministry itself is a complex organization with multiple subordinate agencies, each equipped with dedicated communication departments responsible for disseminating health information within their specific domains (Committee for Medical and Pharmaceutical Control; Committee for Sanitary and Epidemiological Control) [86]. This hierarchical structure, while comprehensive, creates bureaucratic delays that can hinder timely dissemination of critical information.

At the regional level, Kazakhstan’s Territorial Health Departments operate under local authorities, funded through local budgets. They are tasked with local public health engagement, including informing communities about disease outbreaks (Oblast Health Department of Astana; Oblast Health Department of Akmola Oblast). Similar structures exist across other post-Soviet states, with regional health authorities varying by name and degree of autonomy—for example, regional ministries in Russia, oblast health committees in Belarus, and oblast health departments in Central Asian countries. In Azerbaijan, until 2019, rayon-level departments managed district health functions, but these responsibilities have since been transferred to TABIB, the State Agency for Mandatory Health Insurance in Azerbaijan. It is responsible for overseeing and managing healthcare services, implementing health insurance policies, and coordinating health service delivery at the district and national levels [29]. Despite this multi-layered structure, official information regarding health communication strategies, organizational guidelines, or the PRD’s structure remains difficult to access, highlighting transparency issues within the system [45].

Addressing these challenges requires strengthening institutional resources, which are often inadequate. Limited capacity hampers the ability to select appropriate communication channels and develop inclusive, tailored content for diverse populations. Additionally, insufficient investment in professional training impedes the development of skilled health communicators, essential for fostering public trust. Improving these resources is vital, especially during health crises like the COVID-19 pandemic, which exposed the dire consequences of weak institutional capacity for crisis communication and rapid information dissemination [87].

Collectively, these institutional and social weaknesses coalesced into several major communication challenges during and after the pandemic.

#### 3.2.9. Communication Challenges in Post-Soviet States During and Post COVID-19

This section examines health communication challenges, accessing how these obstacles affected public health outcomes and informing lessons for future crises. Key issues include the absence of an institutional communication framework, limited data transparency and accuracy, politicization of measures such as lockdowns and vaccination campaigns, widespread misinformation and disinformation, low public trust and engagement, and drivers of vaccine hesitancy.

##### Data Transparency and Accuracy

Transparent and accurate data are critical during public health crises as they enable individuals to monitor infection rates and hospitalization trends in real time, fostering public awareness and a shared sense of responsibility to curb the virus spread. Moreover, transparency helps build public trust, increasing acceptance of containment measures and vaccination campaigns, ultimately strengthening overall health strategies [70]. However, in post-Soviet states, data transparency is deeply influenced by authoritarian governance structures rooted in the Soviet Semashko health model, which centralized control over healthcare resources, management, and information [33]. This top-down hierarchical system continues to shape health data release and management, especially during crises like COVID-19. For example, in Russia, widespread data manipulation has been reported due to performance-based remuneration systems, with officials sometimes fabricating data or altering cause-of-death records to align with official narratives [88]. During COVID-19, rather than concealing death numbers outright, Russian authorities often manipulated causes of death at registration to align mortality figures with official narratives [88]. During the pandemic, Russian authorities adjusted death classifications rather than outright concealing death figures, but systemic inefficiencies—exacerbated by underfunding after the USSR’s collapse—remain a core issue [33].

Similarly, in Belarus, COVID-19 fatalities were frequently attributed to chronic diseases, and occasional data leaks suggested deliberate underreporting. A previously disclosed document indicated that daily new cases surpassed 1100 by late April, whereas official figures during the first wave did not exceed 1000 [74]. The government presentation in early May 2020 differed from official reports, with prior disclosures suggesting higher case numbers than those officially reported [74].

In Kazakhstan, the early pandemic period was characterized by a lack of reliable information leading to public confusion and the spread of misinformation [78]. The government responded by establishing data centers and an official website, claiming these as the sole trustworthy sources, which limited transparency further and dismissed other information channels [78]. Authorities underscore that transparency enhances trust and is cruel for effective communication, but access to official information remains challenging, complicating efforts to improve openness [45].

The Ministry of Health and its subsidiary organizations are therefore advised to adopt new procedures that make the electronic dissemination of information a priority. Yet, this remains a challenge, as even Salehi et al. [45] reported difficulties in locating documents relevant to the Ministry’s functions during their research. Across Central Asia, the COVID-19 crisis exposed a reluctance among local administrations to report actual infections and mortality data. This often resulted in significant discrepancies between official figures and excess mortality estimates, suggesting possible data manipulation. Studies identified Belarus, Tajikistan, Russia, and Uzbekistan as particularly unreliable health statistics during this period [18,89].

In Armenia, official COVID-19 death statistics significantly undercounted the true toll. By April 2021, reported deaths totaled around 4100, while excess mortality analyses suggested closer to 8300 deaths, indicating undercounting by a ratio of approximately 2.0 [90]. Adjustments for conflict-related deaths, such as those from the Nagorno-Karabakh war, further complicated transparent efforts [90]. After adjusting for war-related fatalities, the level of underreporting remained substantial [90]. Azerbaijan exhibited an even larger discrepancy. While only 3200 COVID-19 deaths were officially reported by February 2021, excess mortality reached nearly 18,000, reflecting an undercount ratio of 5.6 [90]. Glushkova et al. [33] note that rather than deliberate concealment, the lack of accurate data in some cases may reflect ineffective data systems and chronic underinvestment in health infrastructure. Overall, despite some cases where data inaccuracy stemmed from systemic inefficiencies rather than deliberate concealment, the limited transparency and inconsistent data management practices across post-Soviet states severely undermined effective crisis communication and facilitated politicization of public health measures during the pandemic.

##### Politicization of Public Health Measures

The COVID-19 pandemic required governments worldwide to implement public health measures such as lockdowns, movement restrictions, and surveillance systems, but the motives and application of these measures varied significantly across political contexts. While democratic states generally framed these restrictions as temporary efforts to protect public health, authoritarian and hybrid regimes often exploited the pandemic to consolidate power and suppress dissent [91].

##### Politicization of Lockdowns and Surveillance

From the earliest days of the pandemic, Russian authorities used public health restrictions as political tools. In March 2020, only one month into the crisis, enforcement of emergency health regulations was selective, aimed at silencing opposition voices [92]. This trend became more evident by July 2020, when dozens of Moscow residents protesting controversial constitutional reforms were arrested under the guise of pandemic restrictions on gatherings as justification [92]. Even after the pandemic subsided, the legacy of COVID-era restrictions persisted. In March 2023, authorities again arrested individuals protesting the war in Ukraine, citing COVID regulations despite their suspension in other public activities [93,94]. Similarly, in March 2024, the Moscow city government used outdated health measures to block a memorial event for Alexey Navalny, signaling continued willingness to weaponize public health policies to silence dissent long after their health relevance had faded [95].

In Belarus, the pandemic influenced both the 2020 presidential campaign and protest movements. President Lukashenko dismissed COVID-19 as ‘coronavirus psychosis’ and refused to impose lockdowns, while the opposition groups criticized this approach, accusing it of endangering public health. During the pre-election period, mask wearing shifted from a health measure to a symbol of dissent, with demonstrators using masks to express criticism of the government [96].

Across Central Asia, governments leveraged COVID-19 to reinforce their legitimacy as effective managers of the crisis. In ‘performance-based’ authoritarian regimes like Kazakhstan and Uzbekistan, legitimacy relies on visible governance outcomes [97,98,99]. In Kazakhstan, the government used COVID-19 contact tracing to reinforce the invasive surveillance app Sergek (‘Sharp Eye’ in Kazakh), originally intended for traffic monitoring, but expanded for pandemic control [100]. Civil activists who highlighted hospital inadequacies during quarantine faced detention and charges of disseminating false information during the state of emergency [101].

While safeguarding public health is a core governmental responsibility, these cases demonstrate how emergency health measures can be manipulated to serve political interests. Using pandemic restrictions to curb civil liberties and political expression raises critical questions about the balance between health security and democratic freedoms, especially in contexts where governance structures are already fragile or authoritarian. In Armenia, opposition groups, former ruling elites, and civic organizations exploited government missteps, spreading narratives of incompetence and inability to manage the crisis, often driven by clear political motives. The government’s inconsistent messaging created fertile ground for these narratives, politicizing the health response and further eroding public trust [102]. Azerbaijani authorities used COVID-19 restrictions as a tool to suppress dissent, detaining opposition activists and silencing critics under the guise of enforcing lockdown measures. Human Rights Watch reported that at least six activists and a pro-opposition journalist were sentenced to 10–30 days of detention on dubious charges such as violating quarantine rules or disobeying police orders. Most of those targeted had spoken out against poor conditions in state-run quarantine centers or the government’s failure to provide adequate financial support during the crisis [103].

##### Politicization of Vaccination Campaigns

The politicization of public health measures extended beyond lockdowns and surveillance to include vaccination efforts. In Russia, attitudes toward vaccination were highly intertwined with political perceptions. The willingness of individuals’ to receive the Sputnik V vaccine was closely linked to their trust in President Vladimir Putin’s leadership, reflecting how political loyalty and leadership influenced public health behaviors [104]. Moreover, the Kremlin leveraged the Sputnik V vaccine as a geopolitical tool to expand Russia’s global influence [105]. Russian media framed the vaccine as a symbol of national scientific achievement and international solidarity, positioning Russia as a leader capable of saving the world from the pandemic. Simultaneously, these narratives accused Western countries for alleged Russophobia and refused to collaborate, especially when Western governments rejected the Sputnik V vaccine [105]. Unfortunately, this section could not expand due to limited context on vaccine campaigns in other countries and censorship practices.

##### Border Controls and Their Social Implications

The COVID-19 pandemic prompted many governments to impose strict border controls. By spring 2020, approximately 39% of the global population lived in countries that fully closed borders to regular movement, while 91% experienced partial restrictions. Russia and other post-Soviet states generally followed this global trend. However, Belarus took a divergent approach, dismissing the severity of the pandemic and keeping borders relatively open [106]. Nossem [107] reveals that the impact of pandemic-related border controls on social justice was complex. Although mobility restrictions often disproportionately burden the poor, COVID-19 briefly reduced this divide by also limiting the movement of the wealthy as well. Nonetheless, this period of relative equality was offset by a rise in exclusionary and discriminatory practices, as states reinforced power hierarchies under the pretext of safeguarding public health [106].

##### The Spread and Management of Misinformation and Disinformation

Misinformation involves false or misleading beliefs [108] often spread unintentionally by individuals who the information is accurate [109]. Disinformation implies the deliberate spread of false or misleading information aimed at deception [108,109]. Misinformation and disinformation both pose significant challenges to effective public health communication, with contrasting dynamics in democratic and authoritarian contexts. In democratic countries, misinformation often spreads through partisan media, politicians, and social media platforms [108,109]. However, the presence of independent and nonpartisan outlets provides alternative sources for fact-checking and public awareness, helping to curb false narratives [110,111,112]. In authoritarian regimes, where state-controlled media dominate, access to credible information is limited, and governments frequently become primary sources of propaganda and misinformation [113,114].

During the COVID-19 pandemic, misinformation spread rapidly, often outpacing accurate public health guidance. False claims ranged from unproven home remedies to dangerous cures, fueling fear, panic buying, and shortages of essential supplies [115].

The extent and impact of this misinformation varied from region to region, shaped by local contexts and levels of access to reliable information [116]. Both social media and traditional media channels played significant roles in the spread of misinformation during the COVID-19 pandemic, inaccurate treatments, and confusion about health guidelines, with misinformation often circulating through various channels from social media platforms and mainstream news outlets to informal networks [117].

In Russia, the response to COVID-19 demonstrated how state narratives could both suppress and distort information. While the Kremlin officially acknowledged the virus, authorities repressed healthcare workers speaking about equipment shortages and underreported cases [100]. State media downplayed the risks, comparing COVID-19 to seasonal flu or promoting conspiracy theories about its origins [118,119,120].

These messages blended partial truths with distortions, confusing the public and undermining trust [119]. Meanwhile, independent digital outlets provided more accurate information, but the government restricted medical professionals from freely speaking out, requiring approval before media appearances [100]. Similarly, in Belarus, early official messaging was overshadowed by President Lukashenko’s dismissive stance, calling COVID-19 as mere ‘psychosis’ and endorsing unscientific remedies like vodka and saunas [74]. State media amplified these narratives, spreading conspiracy theories and minimizing the virus’s threat to project Belarus as better at managing the pandemic than other countries.

In Central Asia, dire consequences of misinformation and suppressed transparency were evident. Kazakhstan’s government hesitated to share accurate information, which fueled public confusion, underestimation of risks, and the spread of conspiracy theories [78,121]. Major news outlets prioritized attracting traffic over accuracy, further undermining trust and content quality [122]. Turkmenistan entirely denied the existence of COVID-19, removing references to COVID-19 from health materials, intimidating mask users, and promoting herbal remedies without scientific backing [123,124,125]. Despite clear evidence of overwhelmed hospitals and rising mortality rates, the government persisted in denial, restricting public health information and spreading dangerous misinformation [126].

Similarly, Tajikistan refused to acknowledge COVID-19 cases [127] and suppressed independent reporting, blocking news outlets that challenged official narratives [84,128]. Armenia experienced analogous issues, with early officials minimizing the virus’s threat and issuing inconsistent mask mandates that fueled confusion and mistrust. A short-lived ban on non-official health reporting, lifted only under pressure, further damaged information credibility [102].

These dynamics illustrate how weak communication strategies and lack of transparency create fertile ground for misinformation and disinformation, which significantly undermine public trust. This erosion of trust limits the effectiveness of crisis communication efforts and deepens skepticism towards government measures, ultimately hampering compliance and hindering public health efforts [129,130].

##### Lack of Trust in Government and Institutions

The lack of trust in governments and institutions significantly hampers effective public health communication, especially during crises like pandemics. Trust is built through transparent communication and clear explanations of decision-making processes, which foster public confidence, secure cooperation, and support health-protective behaviors [131,132,133]. However, in post-Soviet states, longstanding strained relationships between the state and citizens continue to erode trust in government authority, health systems, and scientific experts [33].

The COVID-19 pandemic exposed a deep crisis of public trust in Russia’s healthcare system and healthcare providers. Contributing factors included a lack of transparency in official statistics, malfunctioning diagnostic tests, and suspended non-COVID care services [100]. While the government acknowledged the pandemic, it simultaneously suppressed negative information, manipulated narratives, and repressed healthcare workers and civic activists voicing concerns. Punitive measures such as selective enforcement of restrictions were perceived as acts of retribution rather than protective measures, fueling social anxiety and further undermining trust [7]. Exemptions granted to state officials and security personnel further alienated the public. In addition, trust in physicians declined due to allegations of hospital-acquired infections, leading to increased lawsuits and prompting some healthcare providers to conceal information to avoid professional repercussions [7].

In Central Asia, distrust in authorities predates COVID-19, rooted in healthcare system challenges within healthcare stemming from the post-Soviet era [134]. In Kazakhstan, inadequate governmental communication and persistent public mistrust were exacerbated during the pandemic by economic hardships, rising unemployment, and inflation, which deepened societal inequalities and eroded confidence in state institutions [41,135]. Conflicting government messages, discrepancies between official statistics, visible healthcare strains, and reliance on delayed reporting contributed to widespread confusion and skepticism [47,78]. Similarly, in Kyrgyzstan, pre-surveys in 2019 already ranked public trust in the Ministry of Health among the lowest, citing corruption and inefficiency as major issues (Figure A3). These perceptions likely contributed to widespread non-compliance with health directives, as evidenced by the surge in COVID-19 cases in Bishkek in mid-2020 despite ongoing awareness campaigns [134].

The lack of trust in authorities across post-Soviet states undermined pandemic response efforts. Weak transparency, politicized decision-making, and systemic corruption created an environment where official health directives often lacked credibility, complicating efforts to promote adherence and effective crisis communication [33]. Restoring institutional trust is essential; research underscores that clear, tailored messaging increases policy acceptance and public confidence [3,136]. Building this trust is crucial for effective health communication during crises and for fostering resilient health systems in the future.

##### Lack of Community Engagement 

Community engagement is the collaborative process that involves people in understanding the risks developing acceptable, practical health response practices. The goal is to empower communities and develop shared leadership throughout the health emergency response cycle [1]. Lack of community engagement has long been a defining characteristic of post-Soviet health systems and health communication practices. Skarphedinsdottir et al. [8] note that the Semashko model fostered a hierarchical approach, where healthcare professionals were viewed as the sole responsible actors for health, leaving little space for patient involvement in care planning or decision-making. This approach has continued to shape governmental health communication strategies, which often lack genuine dialog or participatory mechanisms, even during crises like the COVID-19 pandemic [8]. In Russia, the government established the Stopcoronavirus.rf platform on VK.com to inform citizens about COVID-19 measures. While the platform allowed for public comments, analysis indicates that during the peak phases of the crisis, authorities prioritized deleting comments via automatic moderation rather than engaging with the public, reflecting a censorship-like approach characteristic of authoritarian tendencies [134,137]. Similarly, in Belarus, public participation remains minimal. Despite two decades of independence, there are few patient organizations, and those that exist rarely focus on patient rights or peer education [8,44].

In contrast, some post-Soviet states have attempted to utilize social media for citizen engagement. Azerbaijan’s Ministry of Health engaged the public through its official Twitter account [84], and individual health professionals increasingly used Instagram to raise awareness and educate, although the quality of information shared often needs improvement [138]. In Uzbekistan, the Ministry of Health, along with the Agency of Information and Mass Communications and the Youth Union, created a Telegram channel during the pandemic that attracted over one million subscribers, making it the country’s second most popular channel [139]. Kazakhstan also launched a Telegram channel—the ‘Intersectoral Commission on Preventing the Spread of COVID-19’—to update citizens on the pandemic and government measures. However, as [140] notes, this channel often reported data selectively, posting daily infection numbers only once each morning while updating recovery figures multiple times, thereby fostering a misleadingly optimistic narrative.

While social media and digital platforms were utilized in various contexts, the predominant approach in post-Soviet states remained largely top-down: information was disseminated without substantial dialog or meaningful public participation. This limited the potential to build trust, combat misinformation, and empower citizens to actively participate in public health efforts. Developing more inclusive, participatory communication strategies is essential to enhance public engagement, strengthen trust, and improve health outcomes.

##### Vaccine Hesitancy

Vaccine hesitancy is defined as the reluctance or refusal to vaccinate despite vaccine availability. The WHO regards vaccine hesitancy as a leading global threat, driven by complex interactions among individual beliefs, sociocultural norms, and political factors [139]. This has been a persistent challenge in post-Soviet countries even prior to the COVID-19 pandemic [33]. In the post-Soviet context, vaccine hesitancy is partly rooted in the legacy of hierarchical decision-making structures, where health policies dictated from the top with minimal public input. Such vertical governance structure has fostered deep distrust in health authorities, weakening confidence in vaccination campaigns [33,87]. Despite Russia’s early development of its Sputnik V vaccine, vaccination rates across the region remained notably low, reflecting these entrenched mistrust issues.

Safety concerns have further fueled hesitancy. In Kazakhstan, despite receiving over two million vaccines from Russia in early 2021 and launching its own vaccine in April, widespread doubts persisted regarding vaccine safety, effectiveness, and long-term risks [141]. The rapid development of COVID-19 vaccines raised questions about the rigor of testing, while emerging variants amplified fears about vaccine efficacy [141]. Media narratives shaped a pivotal role in shaping public perceptions. In Kazakhstan, prior to the official vaccination rollout in November 2021, the public had already been exposed to a mixture of accurate and misleading information through social media and news outlets [87]. Many individuals relied on unverified claims about side effects, and government sources were often perceived as untrustworthy, increasing susceptibility to rumors and misinformation [87].

Contrastingly, Kyrgyzstan implemented proactive communication strategies to boost vaccine confidence. The Communication Strategy for Routine Immunization 2018–2020 focused on raising awareness and fostering public commitment through engagement and intersectoral coordination. While routine vaccination coverage remained high in 2018–2019, it declined in 2020 due to the impact of the pandemic [36]. Vaccine hesitancy in post-Soviet states is deeply intertwined with historical governance structures, public mistrust in authorities, safety concerns, and the powerful influence of media narratives. This entrenched highlights broader challenges in public engagement and trust building, underscoring the urgent need for transparent communication and participatory health policy approaches to foster vaccine acceptance and improve health outcomes.

## 4. Discussion

This scoping review aimed to identify the public health communication challenges faced by focusing on nine post-Soviet states—Russia, Belarus, Kazakhstan, Kyrgyzstan, Tajikistan, Turkmenistan, Uzbekistan, Armenia and Azerbaijan—during the COVID-19 pandemic. The key findings are summarized in the comparative table to highlight cross-national patterns in public health communication across the nine post-Soviet states (Table A5).

The study also explores how the historical context of healthcare systems and public health communication practices in these nations has influenced their responses to the crisis. By examining these experiences, the review seeks to derive valuable lessons that can inform and improve future health crisis communication strategies, ensuring more effective responses in the future health crisis. The identified challenges are analyzed through key communication theoretical prisms, including institutional theory, crisis and emergency risk communication (CERC) and trust-building models to connect empirical findings to broader communication scholarship.

### 4.1. Healthcare Systems Challenges

Based on the institutional theory framework, the COVID-19 pandemic revealed how path dependency sustains centralized, hierarchical communication practices in post-Soviet nations. The legacy of the Semashko model, a classic example of institutional persistence, has created a path-dependent system in which health communication strategies are deeply embedded within—and constrained by—institutional structures inherited from the former USSR.

Despite decades of formal healthcare reforms, institutional inertia, driven by underinvestment, limited political commitment, retention of Soviet-era infrastructure, and minimal public engagement, has preserved a top-down communication paradigm [18]. This institutionalized hierarchy leads officials to view citizens as passive recipients rather than engaged partners, reinforcing a monologic communication model rather than fostering dialog [19,43].

Consequently, one cannot analyze the communication failures of the pandemic without first examining the institutional systems that produced them. This politicization of health messaging within these rigid structures further undermined institutional credibility and public compliance [84,86,88]. This was a case especially in authoritarian contexts where communication serves state control rather than public health. Financial constraints, persistent corruption, and outdated facilities [23,28] reflect resource and normative isomorphism, locking these systems into inefficient patterns. The resulting weak public health communication infrastructure—being a product of institutional legacy—challenged effective messaging during COVID-19. Insufficient funding, infrastructure, and public engagement remain critical barriers [23,28,29], underscoring the need for systemic reforms to strengthen health systems and improve crisis preparedness.

To tackle the challenges faced by healthcare systems in nine post-Soviet nations, it is essential for these countries to boost funding for healthcare, improve infrastructure, and promote public engagement as vital measures. These measures could contribute to establishing a strong health system that is well equipped to address public health emergencies and enhance overall health outcomes.

### 4.2. Regional Healthcare Inequality Issues

Post-Soviet countries exhibit stark urban–rural disparities in healthcare access and quality [18]. Rural populations typically offer only basic services and require long travel for specialized care, while urban centers concentrate specialists and advanced technologies [18,41]. Migration of health workers to wealthier regions or abroad, combined with inadequate transport, infrastructure, and uneven regional funding, further depletes rural capacity [18,27,42]. These gaps lead to delayed diagnoses, lower-quality care, and increased strain on remaining staff, perpetuating health inequities [8].

These health inequalities between rural and urban areas challenges might require targeted interventions. Addressing these challenges requires a comprehensive approach, including investments in infrastructure and essential technologies, incentives for healthcare workers to remain in rural areas, and policies aimed at equitable distribution of resources. These also include the implementation of digital health and telemedicine solutions in rural regions and underserved communities. These measures are essential to ensure that all communities have access to equitable healthcare services. Confronting these challenges requires a comprehensive strategy, which includes policies designed to ensure a fair distribution of resources. Such policies should prioritize sustainability to effectively reduce healthcare disparities between rural and urban areas.

### 4.3. Health Communication and Communicators Challenges

Public health communication is undervalued across many post-Soviet states, which limits the development of effective communication strategies and policies [8]. This results in a shortage of qualified health communication professionals, as healthcare PR managers are frequently either untrained specialists or medical staff without communication expertise [8,9]. Trust in governmental institutions and public organizations is strengthened when both information disclosure and decision-making processes are conducted transparently. Within the context of a public health crisis, effective risk communication principles emphasize transparency about the evidence base and decision-making trade-offs [133]. Since trust is grounded in perceptions of expertise, honesty, and care [142], the source credibility model suggests policymakers and communicators must demonstrate reliance on the best available evidence and, where possible, reflect expert consensus in their messaging [143].

Yet, authoritarian health information practices and complex bureaucratic approval processes weaken these initiatives, particularly in times of crisis when timely information is essential [19,78]. Failing to provide clear and consistent information leads to greater confusion, the spread of rumors, and noncompliance—issues that are particularly heightened in emergency situations like COVID-19.

Bridging communication gaps requires coordinated action among all stakeholders. Moreover, the government should play a vital role in ensuring effective health initiatives through active community engagement, and targeted and accessible messaging. While digital channels are essential, their effectiveness depends on pairing them with strategic messaging, media freedom, capacity building, and proactive measures to counter misinformation, ensuring to reach diverse audiences equitably [76]. Importantly, developing health communication strategies and implementation plans should focus on transparency and honesty, while reducing excessive state control. Additionally, establishing and strengthening public health communication infrastructure is also essential, especially to improve messaging channels during crises like COVID-19.

### 4.4. Crisis Communication Challenges During COVID-19

Analyzed through the lens of Crisis and Emergency Risk Communication (CERC), the pandemic response in post-Soviet states revealed critical failures in core principles: transparency, credibility, and coordinated messaging. Gaps in press freedom and data transparency increased public ambiguity and anxiety, creating fertile ground for rumors and misinformation, as predicted by classic communication theory.

These failures manifested directly in the region’s communication strategies. In response to COVID-19, many governments accelerated the adoption of digital tools—social media and virtual campaigns—in an effort to disseminate public health information and combat misinformation [70]. However, gaps in press freedom, data transparency, and institutional capacity limited these efforts [73,81]. In Russia, government control and restrictions hindered accurate reporting, with officials manipulating data [78], and suppressing healthcare workers’ voices [72,100]. Belarus experienced inconsistent messaging and suppression of information, leading to public confusion and mistrust [74]. Kazakhstan’s reliance on official channels of communication often failed to reach all demographics effectively, further limiting engagement [46]. Countries like Tajikistan, Turkmenistan, and Armenia prioritized denial or underreporting, often restricting independent reporting and spreading misinformation about the severity of COVID-19 [119,128]. The widespread dissemination of false cures and conspiracy theories underscores how state-controlled media, acting as a low-credibility source, failed to correct misinformation, allowing alternative narratives to fill the information vacuum.

Misinformation and disinformation flourished in an environment of weak transparency and limited media freedom, thereby undermining public trust. State-controlled media frequently downplayed risks or spread conspiracy theories, thereby reducing the credibility of official communications [74,119]. Social media emerged as a significant source of false cures, misleading information, and panic-inducing rumors, thereby obstructing public health responses. The erosion of trust due to communication failures diminished the effectiveness of crisis management, lowered compliance with health measures, and intensified the spread of misinformation, thereby presenting a significant challenge to effective pandemic management and health communication.

To address these challenges, post-Soviet nations must prioritize the establishment of transparent, inclusive, and adequately resourced health communication frameworks. Strengthening institutional capacity is essential, which includes investing in professional training for public health communicators and establishing clear guidelines for crisis communication that promote transparency and reliability. Enhancing data transparency and ensuring timely, accurate information dissemination are critical for sustaining public trust and combating misinformation. Furthermore, safeguarding press freedom and promoting independent media outlets are vital steps toward creating an environment conducive to truthful information sharing (Figure A4). Governments should also leverage digital platforms effectively by tailoring messages to diverse audiences and collaborating with community leaders and civil society organizations to improve outreach, particularly in rural and marginalized communities. Lastly, international collaboration and adopting best practices in digital communication, including proactive misinformation countermeasures and capacity-building initiatives, can further enhance crisis responses. Ultimately, fostering a culture of openness, accountability, and public engagement will be key to improving crisis communication and ensuring more effective health responses in future emergencies.

### 4.5. Lack of Public Engagement Challenges

Lack of public engagement has characterized post-in post-Soviet health systems, rooted in the hierarchical Semashko model that limited patient involvement in care and decision-making [8]. During the COVID-19 pandemic, government communication strategies largely followed this top-down approach, with minimal genuine dialog. For example, Russia’s Stopcoronavirus.rf platform allowed comments, but authorities often prioritized deleting dissent rather than engaging, reflecting authoritarian tendencies [137]. Similarly, Belarus has had limited patient organizations or participatory mechanisms, hindering public involvement [44].

Some countries attempted to leverage social media for engagement—Azerbaijan’s Ministry and Uzbek authorities used platforms like Twitter, Instagram, and Telegram to reach citizens [84,139]. However, these efforts mostly involved top-down dissemination, with limited avenues for meaningful participation. Kazakhstan’s Telegram updates, for instance, often reported selectively, creating a misleading picture of the pandemic [140]. Overall, the prevalent approach remains one of information delivery rather than dialog [134], highlighting the need for more inclusive, participatory strategies to build trust, combat misinformation, and foster active citizen involvement in public health efforts.

To address these challenges, post-Soviet nations must shift toward more inclusive and participatory communication strategies. This involves creating genuine avenues for citizen engagement, such as establishing patient organizations, facilitating two-way dialog through digital platforms, and involving local communities in health decision-making processes. Governments should prioritize building trust by fostering open, transparent communication that encourages public input and addresses concerns directly. Enhancing health literacy and promoting civic participation can empower individuals to become active partners in health initiatives. Investing in mechanisms for meaningful public engagement will not only strengthen trust and compliance but also improve the overall effectiveness of health responses during crises.

### 4.6. Vaccine Hesitancy and Lack of Trust in Government Issues

The scoping review identifies vaccine hesitancy as a profound challenge in the region, deeply rooted in a crisis of public trust rather than mere misinformation and disinformation [33,87]. Vaccine hesitancy is fundamentally a crisis of institutional trust. Trust-building theory highlights that perceptions of competence, honesty, and benevolence are critical. Historical governance practices eroded these pillars, meaning that even scientifically sound messages were filtered through a lens of deep-seated skepticism. Furthermore, the framing of vaccine messaging played a significant role. In Russia, Sputnik V was framed as a geopolitical achievement, which may have resonated differently with domestic audiences than a frame emphasizing community health and collective responsibility. Understanding how different frames (e.g., individual health vs. public good) interact with existing trust levels is crucial for effective campaign design.

In Kazakhstan, circulating misinformation about vaccine safety and effectiveness, fueled by social media and inconsistent government messaging, further exacerbated hesitancy [80,141]. Conversely, Kyrgyzstan employed proactive communication strategies to improve vaccine confidence, but coverage declined during the pandemic due to broader trust issues [36]. Overall, historical governance practices in post-Soviet nations are closely linked to vaccine hesitancy, public mistrust, safety fears, and the influence of media narratives.

Lack of trust in government and institutions has significantly hindered public health efforts during the pandemic in post-Soviet countries. Given the legacy of hierarchical governance and systemic mistrust, this erosion of confidence was compounded by a history of limited transparency, politicization, and corruption (Figure A3). This distrust hampers effective crisis communication, as authorities often lack credibility, making it difficult to secure public cooperation and promote health-protective behaviors [131,132]. Restoring trust requires transparent, tailored messaging, clear decision-making processes, and efforts to depoliticize health communication. Building institutional credibility is crucial, not only for managing current crises but also for strengthening resilient health systems in the future.

To address these challenges, post-Soviet nations must prioritize transparent, inclusive communication strategies that build public trust and counter misinformation. Enhancing community engagement and involving local leaders can help foster a sense of ownership and confidence in vaccination programs. It is also crucial to improve health literacy by providing clear, accurate information about vaccine safety, efficacy, and the scientific processes behind vaccine development. Therefore, governments should create vaccine messaging, ensuring that scientific evidence backs up projected information. Strengthening existing communication channels, leveraging trusted local voices, and adopting participatory approaches will be essential in overcoming hesitancy and increasing vaccination uptake, ultimately improving public health outcomes.

### 4.7. Theoretical Implications for Health Communication

Empirical studies show that in many post-Soviet nations, despite numerous political, economic, and healthcare reforms, the legacy of the Soviet-era public health communication practices continues to influence their responses to COVID-19. This legacy has shaped communication strategies, eroded public trust, and maintained institutional practices that often exclude meaningful public engagement in risk communication development [8]. Our review confirms that this exclusion had tangible negative consequences during the pandemic, reinforcing the need to strengthen public engagement in future health communication strategies.

Consequently, enhancing public engagement is identified as essential for rebuilding trust, ensuring message relevance, and achieving effective risk communication. Such improvements are vital for supporting the objectives of RCCE-IM [1], strengthening community resilience, and improving public health outcomes in future pandemic preparedness.

The empirical challenges identified in the scoping review (e.g., centralized messaging, misinformation, low trust, and ineffective engagement) directly connect to core communication scholarship. The findings underscore the necessity of moving from a top-down of public communication to context-sensitive models that integrate risk communication principles, trust-building principles, and strategic message framing. Future strategies must be informed by these theories to rebuild credibility and foster public partnership.

## 5. Limitation of the Scoping Review and Conclusions

### 5.1. Limitation of the Scoping Review

This review has several limitations that should be acknowledged. First, despite their shared historical background as part of the USSR, the countries examined vary in their levels of health system organization, and communication strategies and practices. Some have more centralized healthcare systems, while others are less. In addition, although some countries exhibit a more open dialog with society, this transparency often serves as a superficial ‘facade,’ while other nations remain overtly closed systems. As a result, any generalizations about the characteristics of these nine post-Soviet countries should be considered ‘conditional’ and interpreted with caution.

One of the main limitations of this study lies in the scarcity of scholarly and empirical sources on public health communication in post-Soviet countries. Much of the existing literature was produced by international organizations with specific policy agendas or focuses on broader health system reforms rather than communication practices directly. In addition, poor health communication remains a significant problem in many countries as it is often not recognized as one. Discussions of this issue tend to be superficial, requiring the authors to interpret and identify the problem within its specific context. This scarcity of sources posed challenges to conducting a comprehensive comparative analysis and may have resulted in gaps in the evidence base. Despite efforts made to address this by triangulating across international reports, academic publications, and regional policy documents, the limited depth in some areas remains a concern. Thus, the findings should be interpreted with caution. Future research would benefit from more country-level empirical studies and primary data collection to deepen the understanding of public health communication in post-Soviet contexts.

### 5.2. Recommendations

#### 5.2.1. Bridging the Research-to-Practice Gap

While this review maps the systemic challenges, a critical gap remains in the empirical evaluation of communication interventions within the region. Future research must move beyond descriptive analysis to rigorously assess the effectiveness of proposed strategies—such as two-way dialog platforms, digital tools, and specialized training—in real-world settings. Building an evidence base through pilot studies and impact evaluations is essential to inform context-sensitive, evidence-based policies that can genuinely transform public health communication.

#### 5.2.2. Strategic Recommendations for Practice

Based on the findings, several key recommendations can be made to improve public health communication in post-Soviet countries.

- First, it is essential to transition from a one-way information dissemination model to a two-way dialog. Governments should establish transparent channels for feedback and discussion with civil society organizations, community leaders, and the public to foster inclusion and rebuild trust.
- Second, developing clear, pre-established protocols for risk communication is crucial. These protocols should ensure timely, regular, and open data sharing, preventing delays and confusion that erode public confidence. Clarifying roles and responsibilities in advance will also help avoid bureaucratic bottlenecks during emergencies.
- Third, investing in specialized training programs is necessary to produce professionals well versed in both public health and strategic communication [1]. Building this capacity will address the current shortage of qualified experts who can effectively bridge the gap between medical knowledge and public engagement.
- Finally, utilize AI and digital platforms to further streamline information dissemination and ensure accurate, timely updates reach the public. Embracing AI and digital platforms for information dissemination not only streamlines access to critical updates but also ensures that messages are tailored to meet the diverse needs of the community. Health institutions can utilize technology to develop interactive platforms that facilitate real-time feedback and interaction, ultimately fostering a well-informed and resilient society.

Together, these recommendations can form a comprehensive framework for transforming how we navigate future health crises. Implementing these strategies can strengthen crisis communication, build resilience, and restore public trust. Ultimately, this will lead to more informed communities better equipped to respond to future health emergencies.

### 5.3. Conclusions

This scoping review has systematically examined public health communication challenges across nine post-Soviet states during the COVID-19 pandemic. Our findings reveal that despite decades of health system reform, centralized, top-down communication models rooted in the Soviet Semashko legacy remain dominant. This dominance featured often at the expense of transparency, public trust, and community engagement. The COVID-19 response served as a critical evaluation that exacerbated pre-existing inequalities, politicized health messaging, and revealed critical institutional gaps in crisis communication capacity.

In answering our research questions, we identified that: (RQ1) the main challenges included data opacity, politicization of measures, and widespread misinformation; (RQ2) this were profoundly shaped by historical hierarchical governance and persistent urban-rural disparities; and (RQ3) key lessons point to the urgent need for participatory, trust-based communication strategies supported by digital tools and professional training.

Moving forward, policymakers and health authorities in the region must prioritize transparency, institutional capacity building, and meaningful public engagement.

Through this approach, the region can establish robust health systems that are adequately prepared and able to respond effectively to future crises.

## Data Availability

No new data were created or analyzed in this study.

## Appendix A

**Table A1:** PRISMA-ScR Checklist 1.

Section	Item	Prisma-Scr Checklist Item	Page #
**Title**			
Title	1	Identify the report as a scoping review.	1
**Abstract**			
Structured summary	2	Provide a structured summary that includes (as applicable): background, objectives, eligibility criteria, sources of evidence, charting methods, results, and conclusions that relate to the review questions and objectives.	1
**Introduction**			
Rationale	3	Describe the rationale for the review in the context of what is already known. Explain why the review questions/objectives lend themselves to a scoping review approach.	2
Objectives	4	Provide an explicit statement of the questions and objectives being addressed with reference to their key elements (e.g., population or participants, concepts, and context) or other relevant key elements used to conceptualize the review questions and/or objectives.	3
**Methods**			
Protocol and registration	5	Indicate whether a review protocol exists; state if and where it can be accessed (e.g., a Web address); and if available, provide registration information, including the registration number.	3
Eligibility criteria	6	Specify characteristics of the sources of evidence used as eligibility criteria (e.g., years considered, language, and publication status), and provide a rationale.	3–4
Information sources *	7	Describe all information sources in the search (e.g., databases with dates of coverage and contact with authors to identify additional sources), as well as the date the most recent search was executed.	3–4
Search	8	Present the full electronic search strategy for at least 1 database, including any limits used, such that it could be repeated.	4
Selection of sources of evidence	9	State the process for selecting sources of evidence (i.e., screening and eligibility) included in the scoping review.	4–5
Data charting process	10	Describe the methods of charting data from the included sources of evidence (e.g., calibrated forms or forms that have been tested by the team before their use, and whether data charting was done independently or in duplicate) and any processes for obtaining and confirming data from investigators.	5
Data items	11	List and define all variables for which data were sought and any assumptions and simplifications made.	5
Critical appraisal of individual sources of evidence§	12	If done, provide a rationale for conducting a critical appraisal of included sources of evidence; describe the methods used and how this information was used in any data synthesis (if appropriate).	5
Synthesis of results	13	Describe the methods of handling and summarizing the data that were charted.	5–6
**Results**			
Selection of sources of evidence	14	Give numbers of sources of evidence screened, assessed for eligibility, and included in the review, with reasons for exclusions at each stage, ideally using a flow diagram.	5, 41
Characteristics of sources of evidence	15	For each source of evidence, present characteristics for which data were charted and provide the citations.	5, 33–38
Critical appraisal within sources of evidence	16	If done, present data on critical appraisal of included sources of evidence (see item 12).	N/A
Results of individual sources of evidence	17	For each included source of evidence, present the relevant data that were charted that relate to the review questions and objectives.	6
Synthesis of results	18	Summarize and/or present the charting results as they relate to the review questions and objectives.	6–23
**Discussion**			
Summary of evidence	19	Summarize the main results (including an overview of concepts, themes, and types of evidence available), link to the review questions and objectives, and consider the relevance to key groups.	23–27
Limitations	20	Discuss the limitations of the scoping review process.	27–28
Conclusions	21	Provide a general interpretation of the results with respect to the review questions and objectives, as well as potential implications and/or next steps.	29
**Funding**			
Funding	22	Describe sources of funding for the included sources of evidence, as well as sources of funding for the scoping review. Describe the role of the funders of the scoping review.	29

* Where sources of evidence are compiled from bibliographic databases, social media platforms, and Web sites. § The process of systematically examining research evidence to assess its validity, results, and relevance before using it to inform a decision. This term is used for items 12 and 19 instead of “risk of bias” (which is more applicable to systematic reviews of interventions) to include and acknowledge the various sources of evidence that may be used in a scoping review (e.g., quantitative and/or qualitative research, expert opinion, and policy document).

**Table A2:** Search Strategy 1.

Concept	Search Terms (combined with Boolean OR)	Field/Notes
Core Subject	“public health”, “health communication”, “crisis communication”, “risk communication”	Title/Abstract/Keywords
Challenge Focus	“challenge”, “barrier”, “issue”	Title/Abstract/Keywords
Context	“COVID-19”, “pandemic”	Title/Abstract/Keywords
Geographic Scope	“Armenia”, “Azerbaijan”, “Belarus”, “Kazakhstan”, “Kyrgyzstan”, “Russia”, “Tajikistan”, “Turkmenistan”, “Uzbekistan”	Title/Abstract/Keywords
System Context	“Soviet legacy”, “post-Soviet”, “health system”, “Semashko”	Title/Abstract/Keywords
Final Search String	All above concepts combined with Boolean AND: (Core Subject) AND (Challenge Focus) AND (Context) AND (Geographic Scope) AND (System Context)	Title/Abstract/Keywords

**Table A3:** Summary Table of Included Studies.

No	Author(s), Year	Document Type	Country Focus	Key Themes Addressed	Relevant RQ
1	Semenova et al., 2024 [18]	Journal Article	Regional (9 countries)	Historical health systems, inequalities, COVID-19, data transparency	RQ1, RQ2
2	Glushkova et al., 2023 [33]	Journal Article	Regional (9 countries)	Historical health systems, COVID-19, data transparency, public trust	RQ1, RQ2
3	Karpov & Makhnev, 2017 [35]	Journal Article	Regional (9 countries)	Historical health systems	RQ2
4	Karlinsky & Kobak, 2021 [90]	Journal Article	All countries except Turkmenistan	COVID-19, data transparency	RQ1, RQ3
5	Kilani & Georgiou, 2021 [89]	Journal Article	Belarus, Tajikistan, Russia, and Uzbekistan	COVID-19, data transparency	RQ1, RQ3
6	Rechel, B, Sydykova, A et al., 2023 [20]	Journal Article	Central Asia	Historical health systems	RQ2
7	McKee et al., 1998 [40]	Journal Article	Central Asia	Historical health systems	RQ2
8	Moreno-Serra & Wagstaff, 2010 [34]	Journal Article	Central Asia	Health system, reforms, inequalities	RQ2
9	Akhunov, 2020 [81]	Journal Article	Central Asia	COVID-19, health communication, data transparency	RQ1, RQ3
10	Lemon & Antonov, 2021 [91]	Working paper	Central Asia	COVID-19, health communication, politicization, misinformation	RQ1, RQ3
11	Khan, 2021 [121]	Working paper	Central Asia	COVID-19, health communication, misinformation	RQ1, RQ3
12	Torosyan et al., 2008 [26]	Journal Article	Armenia	Historical health systems, reforms, inequalities	RQ2
13	Avakyan et al., 2013 [39]	Journal Article	Armenia	Health system, inequalities	RQ2
14	Breen et al., 2023 [66]	Journal Article	Armenia	Health system, health communication	RQ1, RQ2
15	Barseghyan et al., 2021 [102]	Freedom House Report	Armenia	COVID-19, health communication, politicization, misinformation	RQ1, RQ3
16	United Nations in Armenia, 2022 [48]	Press Release	Armenia	COVID-19, health communication	RQ1, RQ3
17	Permanent Representation of the Republic of Armenia to the Council of Europe, 2020 [82]	Press Release	Armenia	COVID-19, health communication	RQ1, RQ3
18	Graefen & Fazal, 2024 [28]	Journal Article	Azerbaijan	Health system, public engagement	RQ1, RQ2
19	Aliyev, 2021 [83]	Journal Article	Azerbaijan	COVID-19, health communication, public trust	RQ1, RQ3
20	Unlu et al., 2022 [84]	Journal Article	Azerbaijan	COVID-19, health communication, public engagement	RQ1, RQ3
21	Graefen et al., 2023 [138]	Journal Article	Azerbaijan	COVID-19, health communication, public engagement	RQ1, RQ3
22	Quliyeva & Huseynov, 1999 [42]	Journal Article	Azerbaijan	Health system, inequalities	RQ2
23	Ibrahimov et al., 2010 [49]	Journal Article	Azerbaijan	Historical health systems, health communication	RQ2
24	World Bank, n.d. [67]	World Bank Report	Azerbaijan	Health system	RQ2
25	UNICEF in Azerbaijan, 2025 [68]	UNICEF Report	Azerbaijan	Health system	RQ2, RQ3
26	Webb & Gulu, 2024 [29]	WHO Report	Azerbaijan	Health system, reforms, public engagement, inequalities	RQ1, RQ2
27	Human Rights Watch, 2020 [103]	Human Rights Watch Report	Azerbaijan	COVID-19, health communication, politicization	RQ1, RQ3
28	Richardson et al., 2008 [44]	Journal Article	Belarus	Health communication	RQ2
29	Polyakova, 2020 [96]	Journal Article	Belarus	COVID-19, health communication, politicization	RQ1, RQ3
30	Pierson-Lyzhina et al., 2021 [74]	Journal Article	Belarus	COVID-19, health communication, data transparency, misinformation	RQ1, RQ3
31	Skarphedinsdottir et al., 2015 [8]	WHO Report	Belarus	Public communication, public engagement, inequalities	RQ2
32	Richardson et al., 2013 [22]	WHO Report	Belarus	Historical health systems, reforms, inequalities, public engagement	RQ2
33	Webb, 2024 [54]	WHO Report	Belarus	Health system, health communication	RQ1, RQ2
34	Gulis et al., 2021 [21]	Journal Article	Kazakhstan	Health system, reforms, inequalities	RQ1, RQ2
35	Kumar et al., 2013 [24]	Journal Article	Kazakhstan	Health system, reforms	RQ2
36	Iskakov, 2025 [56]	Journal Article	Kazakhstan	Health communication, trust	RQ1, RQ2
37	Bukharbayeva et al., 2022 [87]	Journal Article	Kazakhstan	COVID-19, health communication, public trust	RQ1, RQ3
38	Haruna et al., 2022 [141]	Journal Article	Kazakhstan	COVID-19, health communication, vaccination	RQ1, RQ3
39	Nurumov et al., 2021 [78]	Working paper	Kazakhstan	COVID-19, health communication, public communication	RQ1, RQ3
40	Sharipova & Beissembayev, 2021 [41]	Block Post	Kazakhstan	Health system, inequalities, COVID-19	RQ1, RQ2
41	Amagoh, 2021 [23]	Book	Kazakhstan	Historical health systems, reforms, inequalities	RQ2
42	Kadyrova, 2020 [46]	Book	Kazakhstan	COVID-19, health communication	RQ1, RQ2, PQ3
43	Nair et al., 2020 [135]	Book	Kazakhstan	Policy communication	RQ1, RQ3
44	Abisheva et al., 2020 [75]	Book Chapter	Kazakhstan	COVID-19, health communication, trust	RQ1, RQ3
45	Dulambayeva & Marmontova, 2021 [76]	Book Chapter	Kazakhstan	COVID-19, health communication, public engagement	RQ1, RQ3
46	Kapkyzy, 2021 [77]	Book Chapter	Kazakhstan	COVID-19, health communication, disinformation	RQ1, RQ3
47	Sultanbayeva et al., 2021 [79]	Book Chapter	Kazakhstan	COVID-19, health communication, public engagement	RQ1, RQ3
48	Urpekova et al., 2022 [80]	Book Chapter	Kazakhstan	COVID-19, health communication, framing	RQ1, RQ3
49	Kydyrbaev, 2021 [140]	Book chapter	Kazakhstan	COVID-19, health communication	RQ1, RQ3
50	UNICEF in Kazakhstan, 2022 [55]	UNICEF Report	Kazakhstan	Health communication, vaccination	RQ1, RQ3
51	Dzhalilov et al., 2022 [122]	UNICEF Report	Kazakhstan	COVID-19, health communication, disinformation, vaccination	RQ1, RQ3
52	Salehi et al., 2022 [45]	UNICEF Report	Kazakhstan	Institutional capacity, trust, vaccination	RQ1, RQ3
53	Katsaga & Kulzhanov, 2012 [27]	WHO Report	Kazakhstan	Historical health systems, reforms	RQ2
54	Kulzhanov, 2017 [47]	WHO Report	Kazakhstan	Historical health systems, Health communication	RQ2
55	OECD, 2018 [38]	OECD Review	Kazakhstan	Historical health systems, reforms	RQ2
56	Ministry of Healthcare of Kazakhstan, 2024 [85]	Press Release	Kazakhstan	Historical health systems, Health communication	RQ1, RQ3
57	Committee for Sanitary and Epidemiological Control of the Ministry of Healthcare of Kazakhstan, 2022 [86]	Press Release	Kazakhstan	Historical health systems, Health communication	RQ1, RQ3
58	Bruley & Mamadiiarov, 2020 [134]	Journal Article	Kyrgyzstan	COVID-19, health communication, public trust	RQ1, RQ3
59	Moldoisaeva et al., 2022 [36]	Journal Article	Kyrgyzstan	Health system, reforms, public engagement, health communication	RQ1, RQ2
60	Verma, 2020 [63]	UNICEF Report	Kyrgyzstan	Health communication	RQ1, RQ2
61	Shok & Beliakov, 2020 [7]	Journal Article	Russia	Health communication, COVID-19, trust, manipulation	RQ1, RQ2
62	Sheiman et al., 2018 [17]	Journal Article	Russia	Historical health systems, Soviet legacy	RQ2
63	Popovich et al., 2011 [25]	Journal Article	Russia	Historical health systems, reforms, inequalities	RQ2
64	Antonova, 2009 [51]	Journal Article	Russia	Health communication	RQ2
65	Nikitina & Nikitin, 2015 [52]	Journal Article	Russia	Health communication	RQ2
66	Popov, 2021 [104]	Journal Article	Russia	COVID-19, politicization, vaccination, trust	RQ1, RQ3
67	Kotseva et al., 2023 [105]	Journal Article	Russia	COVID-19, politicization, vaccination	RQ1, RQ3
68	Golunov & Smirnova, 2022 [106]	Journal Article	Russia	COVID-19, health communication	RQ1, RQ3
69	Nisbet & Kamenchuk, 2021 [112]	Journal Article	Russia	COVID-19, health communication, misinformation	RQ1, RQ3
70	Stoycheff et al., 2020 [113]	Journal Article	Russia	Public communication, transparency	RQ1, RQ3
71	Cooper & Fellow, 2020 [118]	Journal Article	Russia	COVID-19, health communication, trust	RQ1, RQ3
72	Sukhankin, 2020 [119]	Journal Article	Russia	COVID-19, health communication, transparency, trust	RQ1, RQ3
73	Tulchinskii, 2020 [120]	Journal Article	Russia	COVID-19, health communication, trust	RQ1, RQ3
74	Pankratov & Morozov, 2021 [71]	Journal Article	Russia	COVID-19, health communication, public engagement	RQ1, RQ3
75	King & Dudina, 2021 [72]	Journal Article	Russia	COVID-19, health communication, data transparency	RQ1, RQ3
76	Litvinenko et al., 2022 [73]	Journal Article	Russia	COVID-19, health communication, politicization	RQ1, RQ3
77	Kofanov et al., 2023 [88]	Journal Article	Russia	COVID-19, health communication, data transparency	RQ1, RQ3
78	Endaltseva, 2020 [19]	Book Chapter	Russia	Actors, fragmentation, media role	RQ1, RQ2
79	Volkovskii & Filatova, 2025 [137]	Conference paper	Russia	COVID-19, health communication, public engagement	RQ1, RQ3
80	Mukhtarova, 2022 [65]	Journal Article	Tajikistan	Health system, public engagement, inequalities	RQ1, RQ2
81	Boboyorov, 2021 [128]	Book chapter	Tajikistan	COVID-19, health communication, misinformation, transparency	RQ1, RQ3
82	Sodiqova et al., 2025 [64]	WHO Report	Tajikistan	Health communication	RQ1, RQ2
83	Robinson et al., 2024 [32]	WHO Report	Tajikistan	Health system, reforms, inequalities	RQ1, RQ2
84	World Bank, 2022 [31]	World Bank Report	Tajikistan	Public Expenditure (health system)	RQ2
85	Yaylymova, 2020 [123]	Journal Article	Turkmenistan	COVID-19, health communication, misinformation, transparency	RQ1, RQ3
86	Hashim et al., 2022 [126]	Journal Article	Turkmenistan	COVID-19, health communication, misinformation, transparency	RQ1, RQ3
87	Ahmedov et al., 2015 [37]	Journal Article	Uzbekistan	Health system, reforms	RQ2
88	Cancarini, 2020 [58]	Journal Article	Uzbekistan	COVID-19, health communication	RQ1, RQ2
89	Vikhrov et al., 2021 [139]	Journal Article	Uzbekistan	COVID-19, health communication, public engagement	RQ1, RQ3
90	Lemon, 2019 [99]	Working paper	Uzbekistan	COVID-19, health communication, politicization	RQ1, RQ3
91	Robinson & Yin, 2024 [30]	WHO Report	Uzbekistan	Health system, reforms, public engagement	RQ1, RQ2
92	Robinson, 2023 [57]	WHO Report	Uzbekistan	Health system, health communication	RQ1, RQ2
93	United Nations, 2024 [59]	UN Report	Uzbekistan	Health system	RQ2
94	UNICEF in Uzbekistan, n.d. [62]	UNICEF Report	Uzbekistan	Health system	RQ2
95	Ministry of Health of the Republic of Uzbekistan, n.d. [60]	Press Release	Uzbekistan	Health system	RQ2

**Table A4:** Health Expenditure (%) of GDP.

Countries	2020	2021	2022
Armenia	12.24	12.32	9.96
Azerbaijan	5.85	4.89	3.98
Belarus	6.41	6.57	6.56
Kazakhstan	3.75	3.92	3.74
Kyrgyzstan	4.95	5.35	4.92
Russia	8.04	6.98	6.92
Tajikistan	8.89	8.38	7.63
Turkmenistan	5.57	5.49	5.37
Uzbekistan	6.71	7.70	7.36

This chart was created with data gotten from the World Bank [144].

**Table A5:** Comparative Summary of Public Health Communication Challenges in Nine Post-Soviet Countries.

Country	Health Communication System	Crisis Communication (COVID-19)	Transparency & Data Issues	Public Trust & Misinformation	Key Structural Challenges
Russia	Centralized; state-controlled; grassroots mediators exist but constrained.	Digital adoption increased; communication reformatted for remote interaction.	Data manipulation (cause-of-death reclassification); underreporting.	Low trust; state media spread conspiracies; healthcare voices suppressed.	Highly centralized hierarchy; pharmaceutical marketing dominance; politicization of measures.
Belarus	Highly centralized, top-down; minimal public or patient involvement.	Inconsistent; official messaging dismissed threat (“psychosis”).	Data leaks suggested underreporting; deaths attributed to chronic diseases.	Public confusion and mistrust; leader’s dismissive stance fueled skepticism.	Soviet-era paternalistic style; lack of patient organizations; weak public engagement.
Kazakhstan	Ministry-led; multi-platform use; regional departments for localization.	Proactive campaigns; used websites, social media, Telegram.	Early lack of reliable info; later official portals were sole “trusted” sources.	Public mistrust; bureaucratic language limited reach; mixed messaging.	Lack of overarching strategy; staffing shortages in PR; urban-rural digital divides.
Uzbekistan	Multi-actor (gov’t, int’l orgs, NGOs); emphasis on necessity of digital health/telemedicine.	Combined strict measures with info campaigns; large Telegram channel for updates.	Discrepancies between official stats and excess mortality estimates.	Fragmented coordination low trust; digital access gaps in rural areas.	Bureaucratic delays; donor-driven short-term projects; weak crisis preparedness.
Kyrgyzstan	Ministry of Health oversees health communication initiatives, supported by specialized entities such as the Republican Center for Health Promotion and Mass Communication.	Proactive communication strategies to boost vaccine confidence	Pre-pandemic surveys showed very low trust in Ministry of Health.	Preexisting distrust reduced compliance despite awareness campaigns.	Limited research on communication effectiveness; reliance on international support.
Tajikistan	Ministry-led; relies on community members as health communicators in remote areas.	Restrictive; delayed acknowledgment of cases; limited data sharing.	Significant underreporting; denial of outbreaks during election period.	Misinformation spread due to lack of official transparency; community communicators often untrained.	Weak infrastructure; rural access barriers; reliance on informal networks.
Armenia	Multi-actor (gov’t, NGOs, int’l); digital and telemedicine focus.	Centralized under Commandant’s Office; restricted independent reporting.	Significant undercounting of COVID-19 deaths (~2:1 ratio vs. excess mortality).	Opposition politicized government missteps; inconsistent messaging eroded trust.	Weak horizontal/inter-departmental coordination; focus on emergencies over routine messaging.
Azerbaijan	Managed by Ministry and PHRC; NGO involvement (AHCA).	Official websites/social media promoted measures; WHO trained journalists to counter misinformation.	Large data discrepancy (undercount ratio ~5.6); low transparency.	Low public trust impeded compliance; fragmented coordination and unclear roles.	Rural digital divides; inconsistent messaging; weak monitoring/evaluation systems.
Turkmenistan	Highly restrictive; state-controlled denial of health crises.	Denied domestic cases; removed “COVID-19” from materials; intimidated mask users.	No official data; independent reports described severe shortages and overwhelmed hospitals.	State-sponsored misinformation (e.g., promoted herbal remedies); complete lack of public trust.	Total lack of transparency; health communication used as propaganda; no independent media.
